# The characterization of *Klebsiella pneumoniae* associated with neonatal sepsis in low- and middle-income countries to inform vaccine design

**DOI:** 10.1038/s42003-025-08258-7

**Published:** 2025-06-09

**Authors:** Francesca Nonne, Mariagrazia Molfetta, Gianina Florentina Belciug, Martina Carducci, Virginia Cianchi, Casey Zakroff, Salvatore Durante, Caroline Zellmer, Stephen Baker, Thomas D. Stanton, Kathryn E. Holt, Kelly Wyres, Neil Ravenscroft, Gianmarco Gasperini, Omar Rossi, Carlo Giannelli, Francesco Berlanda Scorza, Francesca Micoli

**Affiliations:** 1 GSK Vaccines Institute for Global Health (GVGH), Siena, Italy; 2 Department of Biomedical Sciences, Humanitas University, Milan, Italy; 3 IRCCS Humanitas Research Hospital, Milan, Italy; 4 GSK, Siena, Italy; 5 The Department of Medicine, University of Cambridge, Cambridge, United Kingdom; 6 A*STAR Infectious Diseases Labs (A*STAR IDL), Agency for Science, Technology and Research (A*STAR), Singapore, Singapore; 7 Department of Infectious Diseases, School of Translational Medicine, Monash University, Melbourne, VIC Australia; 8 Centre to Impact AMR, Monash University, Clayton, VIC Australia; 9 Department of Infection Biology, Faculty of Infectious and Tropical Diseases, London School of Hygiene and Tropical Medicine, London, UK; 10 Department of Chemistry, University of Cape Town, Cape Town, South Africa

**Keywords:** Bacterial infection, Polysaccharides

## Abstract

*Klebsiella pneumoniae* is the leading cause of neonatal sepsis, strongly associated to antimicrobial resistance, with no vaccine available. K-antigens (KAg) have been identified as potential targets, but their diversity makes vaccine development challenging. Alternatively, the use of subcapsular O-antigens (OAg) raises questions about antibodies accessibility. We characterized clinical isolates from the BARNARDS study, designed to identify the burden of neonatal sepsis in low-middle income countries. Genomic prediction was verified through structural analysis of polysaccharides. Antibodies generated against common KAg and OAg bound all homologous organisms, regardless of specific polysaccharide structural features. Interestingly, anti-KAg antibodies exhibited bactericidal activity against a comparable number of isolates as anti-OAg antibodies. There was no association between polysaccharide characteristics and *K. pneumoniae* susceptibility to killing. Antibody cross-reactivity among different KAg was observed, together with extensive cross-reactivity among OAg antibodies. This study aids in defining an optimal vaccine composition to prevent neonatal sepsis caused by *K. pneumoniae*.

## Abstract (web summary)

A potential correlation between polysaccharide structural features and the ability of corresponding antibodies to bind and kill in vitro a large panel of *Klebsiella pneumoniae* clinical isolates, supporting the development of a multivalent vaccine.

## Introduction

*Klebsiella pneumoniae* (*K. pneumoniae*) is the leading etiological agent of neonatal sepsis globally^1,2^, and has been classified as a critical-priority antimicrobial resistant (AMR) pathogen by World Health Organization (WHO)^3^. The organism is the second leading cause of death attributable to AMR globally, and the leading cause of death in sub-Saharan Africa^4^. The rapid emergence of AMR, particularly extended-spectrum beta-lactamase (ESBL) and carbapenemase-producing organisms, limits therapeutic options, leading to increased mortality^5^.

The Child Health and Mortality Prevention Surveillance (CHAMPS) Network generated data on mortality in children aged under five years and stillbirths in seven countries in sub-Saharan Africa and South Asia between 2016 and 2020; *K. pneumoniae* was among the most frequent pathogens associated with neonatal deaths^6,7^. Similarly, the Burden of Antibiotic Resistance in Neonates from Developing Societies (BARNARDS) study, a multi-center study across seven sub-Saharan African and South Asian countries between 2015 and 2017, found *K. pneumoniae* to be the leading cause (24.9%) of neonatal sepsis, with >80% of Gram-negative bacteria resistant to third generation cephalosporins and 13–15% resistant to carbapenems^1^. The global neonatal sepsis observational cohort study (NeoOBS), which examined sepsis and antimicrobial usage in 11 countries from 2018 to 2020^8^, determined that 37% of the Gram-negative organisms were *K. pneumoniae*, most of which were resistant to WHO-recommended regimens and carbapenems.

A maternal vaccine against *K. pneumoniae* is highly warranted. A safe and effective vaccine delivered to women in the second or third trimester of pregnancy, which results in transplacental transfer of protective antibodies to the fetus, could reduce the risk of invasive disease in young infants in the neonatal period and first few months of life, and also reduce antimicrobial usage. A modeling study assuming 70% population coverage calculated that a *K. pneumoniae* vaccine for pregnant women may avert ~80,000 deaths and ~400,000 cases of neonatal sepsis, having the greatest impact in sub-Saharan Africa and South Asia^9^. Despite significant effort, no vaccines against *K. pneumoniae* have yet been licensed, but several vaccines are currently under preclinical development, with a few tested in clinical trials^10,11^.

Two types of polysaccharides (PS) are presented on the surface of *K. pneumoniae* and have been proposed as potential antigens for vaccine design: capsular polysaccharides, historically known as K-antigens (KAg), and O-antigens (OAg)^12^. KAg are complex acidic polysaccharides frequently consisting of repeating units of three to six sugars, one of which is typically a uronic acid^13^. KAg has substantial diversity, with nearly 80 distinct serotypes identified by sero-immuno assays, and additional 90 proposed based on unique gene sequences in the biosynthesis locus^14,15^.

In contrast to the large number of KAg serotypes, less OAg gene clusters have been identified to date^15–18^. Among them, O1 and O2 OAg share the [→3)-α-D-Gal*p*-(1 → 3)-β-D-Gal*f-*(1 → ] repeating unit, also known as Galactan-I (Gal-I), defining serotype O2v1 (or O2a). This backbone can be further decorated through the addition of an α(1 → 4)-linked galactose side chain, which modifies Gal-I to Galactan-III (Gal-III) and converts serotype O2v1 into O2v2 (or O2afg). Furthermore, the addition of a capping polysaccharide composed by the [→3)-α-D-Gal*p*-(1 → 3)-β-D-Gal*p*-(1 → ] repeating unit, also known as Galactan-II (Gal-II), converts serotype O2v1 or O2v2 into serotype O1v1 or serotype O1v2, respectively. O3 (including the related serotypes O3a and O3b) and O5 are instead composed of D-mannoses (Man). O4 has a different repeating unit structure, made of ribofuranose (Rib*f*) and galactose (Gal) sugars.

Several studies have shown the ability of anti-KAg antibodies to confer protection against *K. pneumoniae* in animal models of infection^10,11^, while some doubts have been raised about the ability of anti-OAg antibodies to induce protection. Indeed, while OAg-based vaccines have shown protection in some mouse models of invasive *Klebsiella*, other studies have indicated that the presence of KAg can interfere with the ability of OAg antibodies to bind and kill *K. pneumoniae*^19–21^.

The aim of this work was to characterize a large library of isolates from the BARNARDS study and identify polysaccharide structural features. We developed a high-throughput purification method to obtain sufficient KAg and OAg material for analysis. Hyperimmune sera were generated in rabbits against glycoconjugates prepared using prevalent KAg and matched OAg to generate data on antibody binding and functional activity of respective antibodies against the panel of *K. pneumoniae* isolates. These data are key to confirm the sero-epidemiology specific to neonatal sepsis in LMICs and to better understand the potential for capsular and sub-capsular antibodies and may ultimately contribute to an optimal vaccine formulation to prevent neonatal sepsis caused by *K. pneumoniae*.

## Results

### High level characterization of *K. pneumoniae* polysaccharides in a large panel of isolates from LMICs

**Fig. 1:**
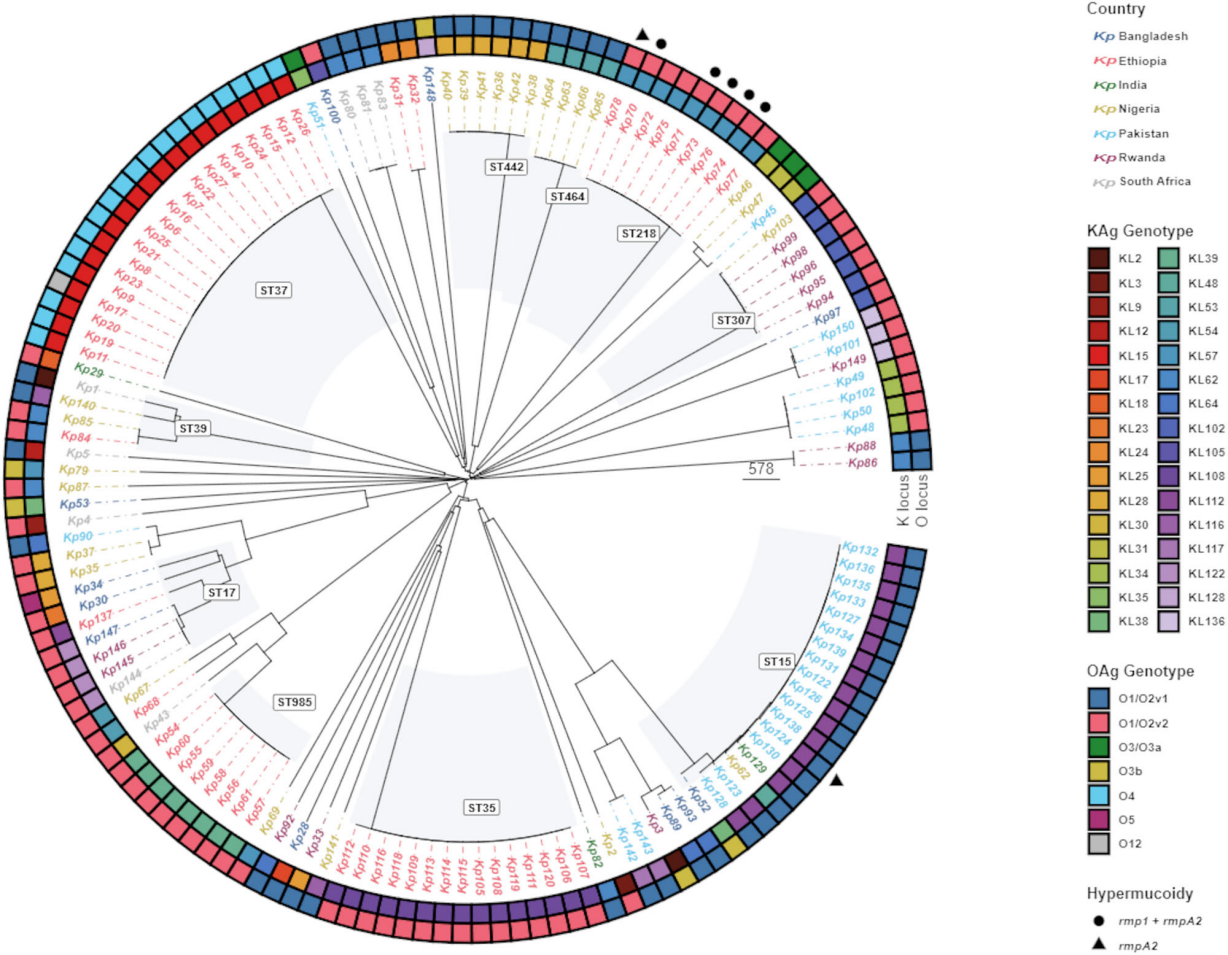
Phylogenetic distribution of K and O loci. Neighbour-joining tree constructed from *n* = 143 genome assemblies of *K. pneumoniae* isolates used in this study (excludes genomes that failed QC and those that had Untypeable K and/or O loci), using PathogenWatch core gene single nucleotide differences^25^ Tree tips are labelled by the identifiers used in this study and are coloured by the country of origin of the corresponding isolate. Clades of 4 or more isolates of the same 7-gene multi-locus sequence type as determined by Kleborate^40^ are highlighted. Coloured rings represent the KAg and OAg genotypes (K- and O-loci) respectively, determined by Kaptive^42^, and isolates are marked with a circle if they are positive for the and rmpADC locus (lineage rmp1) plus the rmpA2 gene, or a triangle if they are positive for just the rmpA2 gene. The tree was plotted using the ggtree R package^43^.

One hundred and fifty *K. pneumoniae* associated with neonatal sepsis from the BARNARDS study were analyzed in this study. One-hundred and forty-four isolates had matched whole-genome sequences^1^, 143 of which passed quality control and included a diverse range of AMR *K. pneumoniae* lineages. According to the genomic analysis they represented 32 different K loci (KL) associated with distinct predicted K-serotypes, and O loci associated with 7 different O-serotypes (Fig. 1). In general, strains that formed closely related clades shared the same K and O loci. However, there were also examples of distantly related strains that shared the same loci, e.g. O1/O2v1 and O1/O2v2 loci were present in multiple unrelated clades (Fig. 1). Each KAg type was predominantly associated with one specific OAg serotype. Overall, 19/20 KL15 were O4 associated, with O4 not found with any other KAg. A very small number of strains carried the *rmp*A2 and/or *rmp*ADC hypermucoidy loci that have previously been shown to result in capsule hyperexpression and/or increased capsule chain length^22,23^; however there were too few strains here for robust statistical comparison (Fig. 1).

*K. pneumoniae* isolates were grown in laboratory conditions selected to maximize KAg and OAg production. A high-throughput method for polysaccharide extraction and purification was established, and isolates were characterized for polysaccharide molecular weight, structural monosaccharide composition, and quantity of sugar produced (Supplementary Fig. 1)^24^. Chemical structure characterization was performed to validate KAg and OAg type prediction from genomic information.

**Fig. 2:**
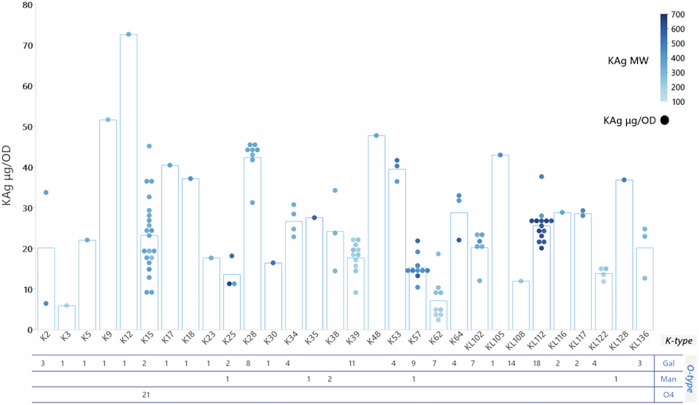
KAg structural features—polysaccharide amount and size. Association between KAg and OAg is reported for all isolates analyzed. Amount of KAg produced (normalized per optical density (OD)) is reported on the y-axis (single dots corresponding to each isolate) and the average for each K-serotype is represented by the bars. Color codes are associated to KAg molecular weight (MW) in kDa, ranging from 149 to 662. Strains not expressing the KAg are included in the O-type numbers but do not have a molecular weight dot in the graph.

Among the 116 isolates for which KAg structures were chemically determined and genomic predictions were available, 108 (93%) were concordant (Supplementary Table 1). Among the 141 isolates for which OAg composition was chemically determined and genomic predictions were available, 139 (99%) were concordant (Supplementary Table 1). A summary of structural KAg characteristics obtained for each isolate is reported in Fig. 2.

**Fig. 3:**
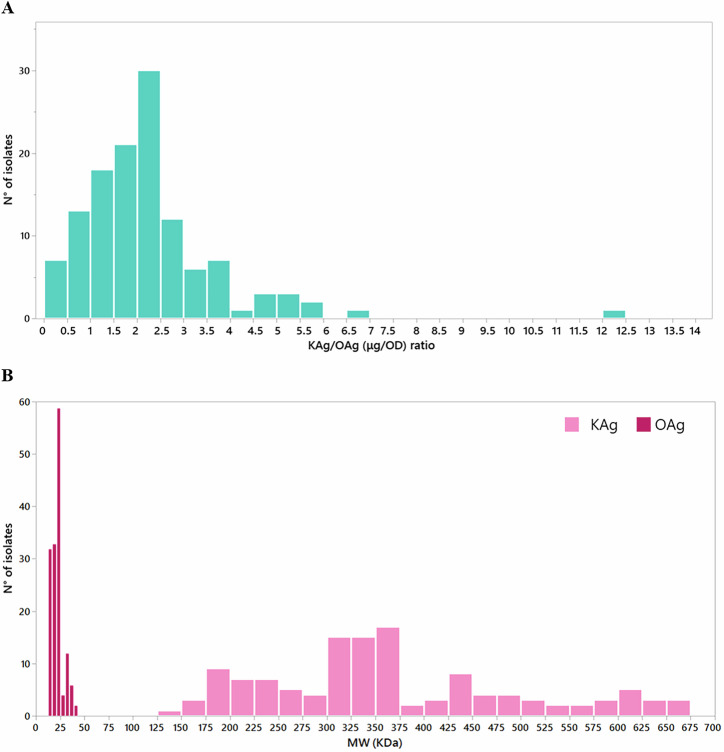
Structural features of KAg and OAg in the panel of isolates characterized. KAg to OAg amount ratio for all the isolates characterized (**A**). Molecular weight (MW) of KAg and OAg extracted from the 150 isolates (**B**).

Some of the isolates did not express the KAg (e.g., 13 out of 14 KL108 isolates, Fig. 2) in the growth conditions tested. No clear correlation was determined between KAg quantity and size (Supplementary Fig. 2). For some serotypes, the results were homogeneous (e.g., K39, K28, KL112), for others variation was observed. For example, all K15 isolates were remarkably similar in terms of MW (around 350 kDa) but differed in the amount of produced KAg. Alternatively, the K57 isolates produced similar amounts of polysaccharide (~10–20 ug/OD) but differed in polysaccharide length. Notably, all KL112 isolates expressed very long KAg (>550 kDa). Overall, KAg exceeded OAg production (Fig. 3A), and KAg (~150–660 kDa) was much longer in length than OAg (~10–45 kDa) (Fig. 3B). The ratio of KAg/OAg amount most commonly fell around 2 (Fig. 3A), and the ratio of KAg/OAg size most commonly fell around 20 across strains (Supplementary Fig. 3), suggesting some sort of selected relationship between these structures.

### Synthesis of glycoconjugates and generation of hyperimmune sera

Selected KAgs, corresponding to common serotypes reported from LMICs^1,25,26^, and associated OAg, were purified at larger scale^24^, and corresponding glycoconjugates were prepared for generation of hyperimmune sera in rabbits. Random activation of the polysaccharide chains by CDAP chemistry^27^ was used both for KAg and OAg; CRM_197_ was selected as carrier protein. Conjugate formation was verified by HPLC-SEC analysis in comparison to free protein (data not shown). Supplementary Table 2 reports saccharide to protein ratios of the resulting purified glycoconjugates. Rabbits immunization resulted to elicit high anti-antigen specific IgG (Supplementary Fig. 4A), and pooled sera were assessed for their ability to kill a panel of selected strains expressing homologous KAg and OAg in a luminescent-based Serum bactericidal assay (L-SBA)^28,29^ (Supplementary Fig. 4B).

### Antibody binding and functionality against isolates sharing homologous polysaccharides

Pooled sera generated in rabbits (both anti-KAg and anti-OAg) were tested for their ability to bind the homologous bacteria from the BARNARDS study by Flow Cytometry (FC) analysis. Sera were able to bind capsular (KAg) and sub-capsular (OAg) antigens in all isolates tested (Supplementary Tables 3–4). To be noted that binding of anti-OAg antibodies was higher to non-capsulated strains (data not shown). We then assessed how binding may translate to the ability of antibodies to kill strains with a homologous KAg in L-SBA. The same isolates were also tested in L-SBA with sera against the OAg that they displayed.

**Fig. 4:**
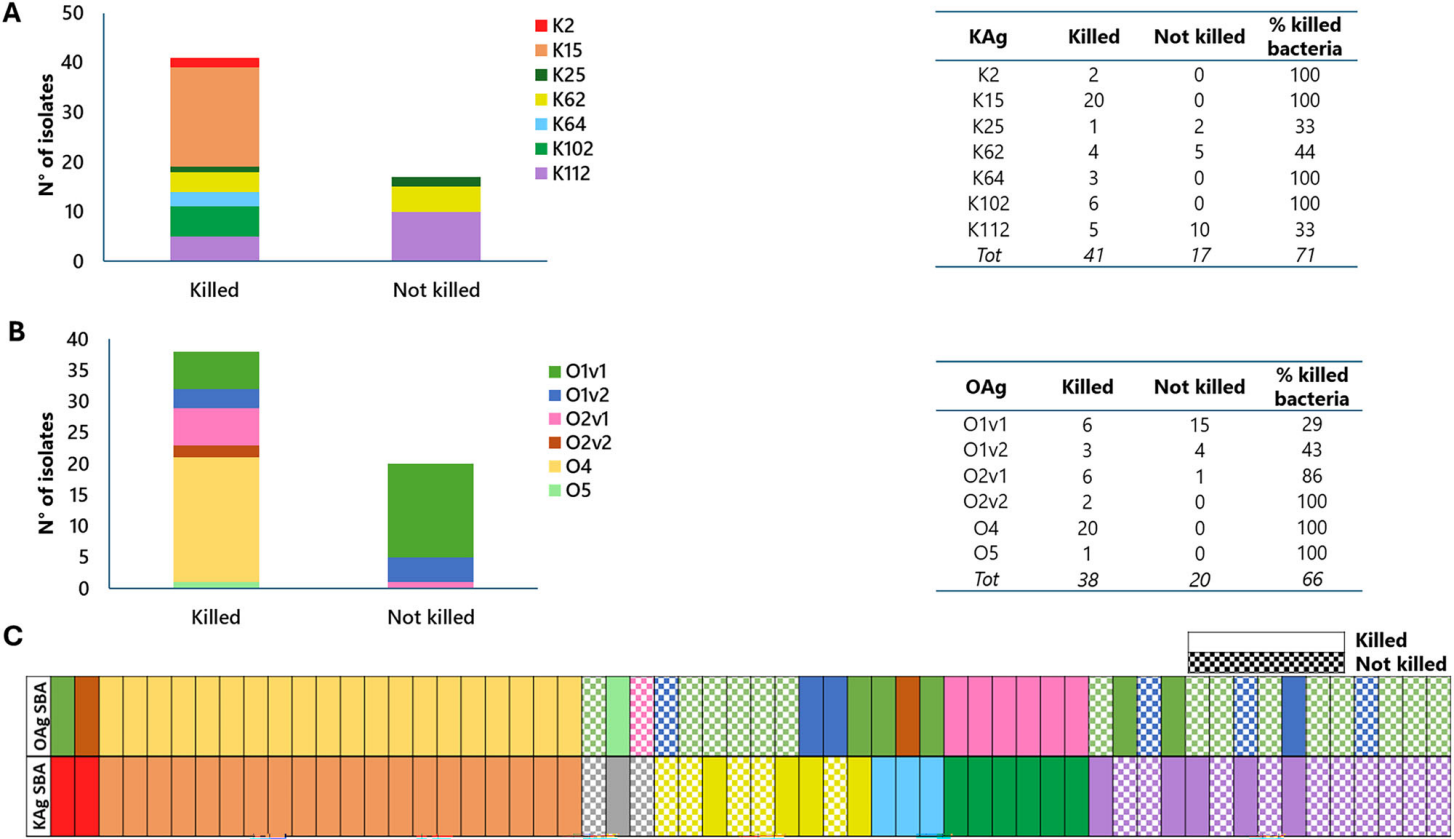
Bacterial susceptibility to killing by homologous sera in L-SBA. Number of isolates killed in L-SBA by anti-KAg (**A**) or anti-OAg (**B**) antibodies. Map of L-SBA results, indicating isolates killed or not by homologous anti-KAg or anti-OAg antibodies (**C**). % Killed bacteria refers to number of isolates for which an IC50 (the reciprocal of serum dilution able to kill 50% of the bacteria) > 10 (no killing) was assigned.

Out of the 58 isolates tested, 41 (71%) were killed by anti-KAg antibodies (Fig. 4A), and 38 (66%) were killed by anti-OAg antibodies (Fig. 4B). In total 36/58 (62%) of isolates were killed by both anti-KAg and anti-OAg antibodies, while 15/58 (25%) of organisms were resistant to killing by anti-KAg and anti-OAg antibodies. SBA was also investigated by mixing anti-KAg and anti-OAg antibodies, confirming no killing (Supplementary Fig. 5). From the 20 isolates that were resistant to anti-OAg antibodies, 5 were killed by anti-KAg antibodies (25%), while 2/17 strains not killed by anti-KAg antibodies were killed by anti-OAg antibodies (12%) (Fig. 4C). KAg table in Fig. 4A shows the proportion of isolates killed for each K-serotype. For the OAg, Gal-based serotypes (O1v1, O1v2, O2v1, O2v2) were assigned according to FC and SBA results obtained with serotype-specific sera. Isolates resistant to anti-OAg antibodies most expressed the O1v1 or O1v2 type OAg (19 of 20, Fig. 4B).

**Fig. 5:**
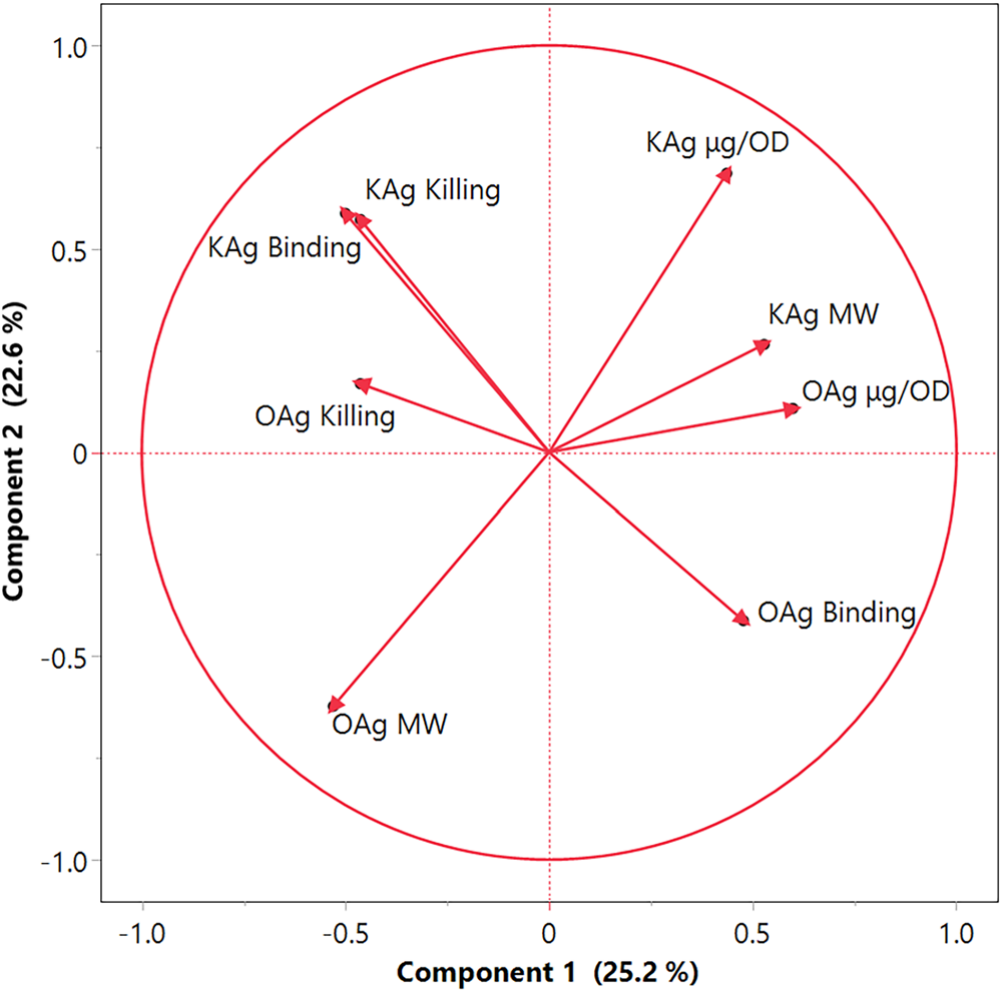
Principal component analysis correlating antibody binding/killing to sugar structural features (amount and size). MW stands for molecular weight; killing refers to SBA titers; binding refers to mean fluorescence intensity (MFI) by FC.

A principal component analysis was used to correlate antibody binding/killing results to structural features of the polysaccharides (quantity and length). The analysis was able to explain 48% of data with the first two components (Fig. 5). Serum bactericidal titers elicited by anti-KAg antibodies correlated with bactericidal titers elicited by anti-OAg antibodies (“KAg Killing” and “OAg Killing”, Fig. 5). Most bacteria were killed both by anti-KAg and anti-OAg antibodies (Fig. 4B). Notably, KAg antibody binding strongly correlates with SBA titers (“KAg Binding” and “KAg Killing”, Fig. 5). SBA titers elicited by anti-OAg antibodies anti-correlate with OAg antibodies binding (“OAg Killing” and “OAg Binding”, Fig. 5) and OAg antibodies binding anti-correlates with KAg antibodies binding (Fig. 5). No clear correlation was observed between binding/killing and sugar structural features, both for KAg and OAg.

### Antibody binding and functionality against isolates sharing heterologous KAg

**Fig. 6:**
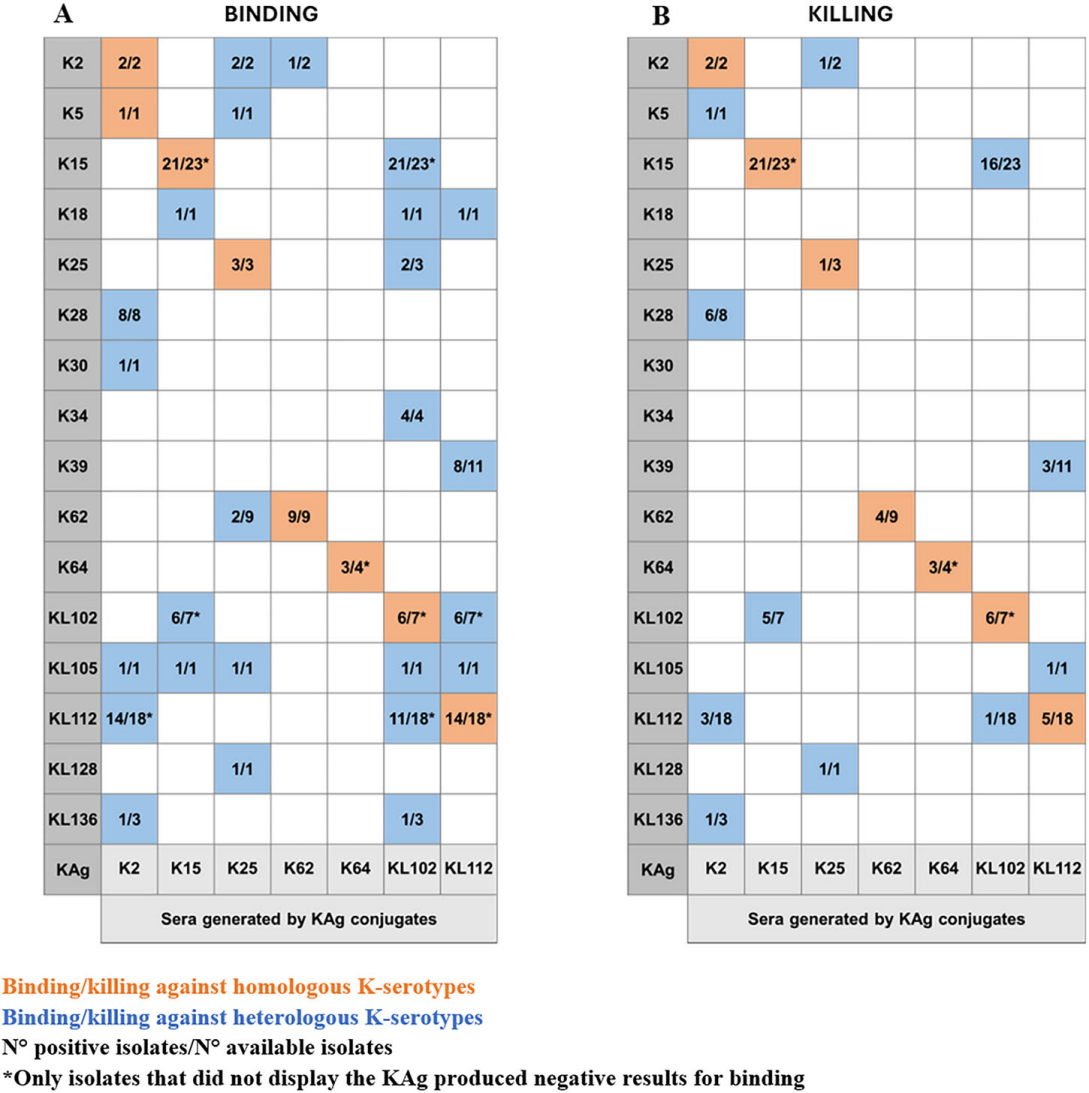
Bacterial binding and killing by homologous and heterologous KAg sera. Hyperimmune anti-KAg sera tested for ability to bind (**A**) and kill (**B**) the entire panel of isolates displaying homologous and heterologous K-serotypes. Only K-serotypes showing cross-binding are reported and were tested for SBA.

Pooled sera were then tested against the entire panel of isolates to investigate their ability to bind organisms with heterologous KAg (Supplementary Table 3). We observed some cross-reactivity by FC (Fig. 6). Only K64 serum demonstrated an ability to bind exclusively its homologous K-serotype. For all others, cross-binding was verified for at least one additional serotype among those evaluated. Anti-K2, KL102 and KL112 sera showed cross-binding against the highest number of heterologous isolates (Fig. 6A, Supplementary Fig. 6). The *K. pneumoniae* demonstrating cross-reactive binding were evaluated in SBA to verify the ability of sera to kill isolates displaying heterologous KAg. Almost half of heterologous reactivity also resulted in killing (Fig. 6B). K2 serum shows binding and bactericidal activity to K5, K28, KL112 and KL136 strains; K15 serum to KL102 only; K25 serum to K2 and KL128; KL102 serum to K15 and KL112 and finally KL112 serum to K39 and KL105.

**Fig. 7:**
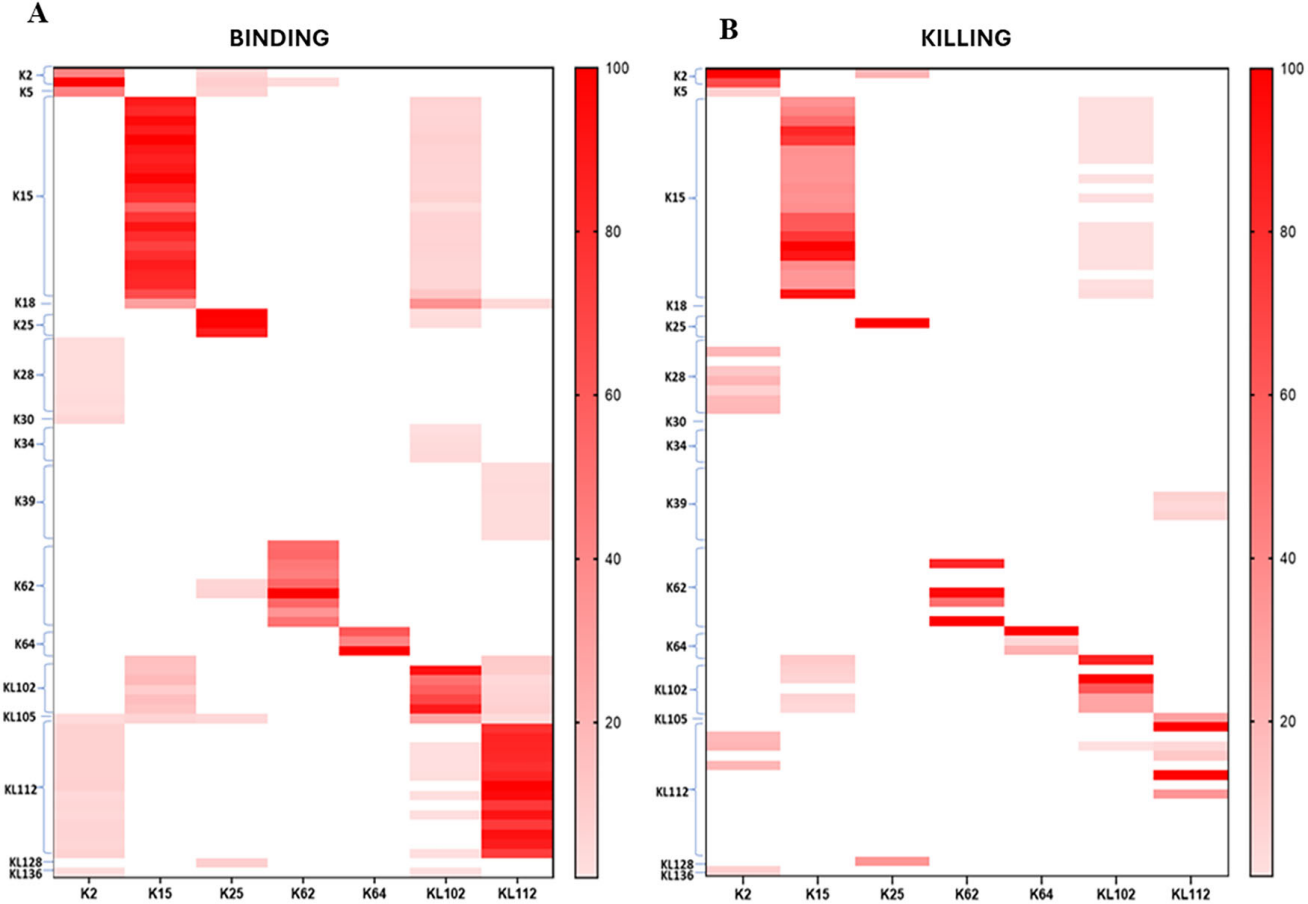
Heterologous cross-reactivity. FC (**A**) and SBA (**B**) of sera listed on the bottom against panel of heterologous strains listed on the y-axis. Isolates that gave positive signals in FC were tested by SBA. FC: MFI increases with respect to negative control normalized to 100% on homologous strains. SBA: IC50 titers normalized to 100% on homologous strains.

However, by comparing the binding of sera against homologous or heterologous KAg, we observed that cross-reactive binding was of lower intensity for heterologous isolates compared to the homologous ones (Fig. 7A, Supplementary Fig. 6, Supplementary Table 3). Comparably, SBA titers were also lower against heterologous compared to homologous isolates (Fig. 7B).

**Fig. 8:**
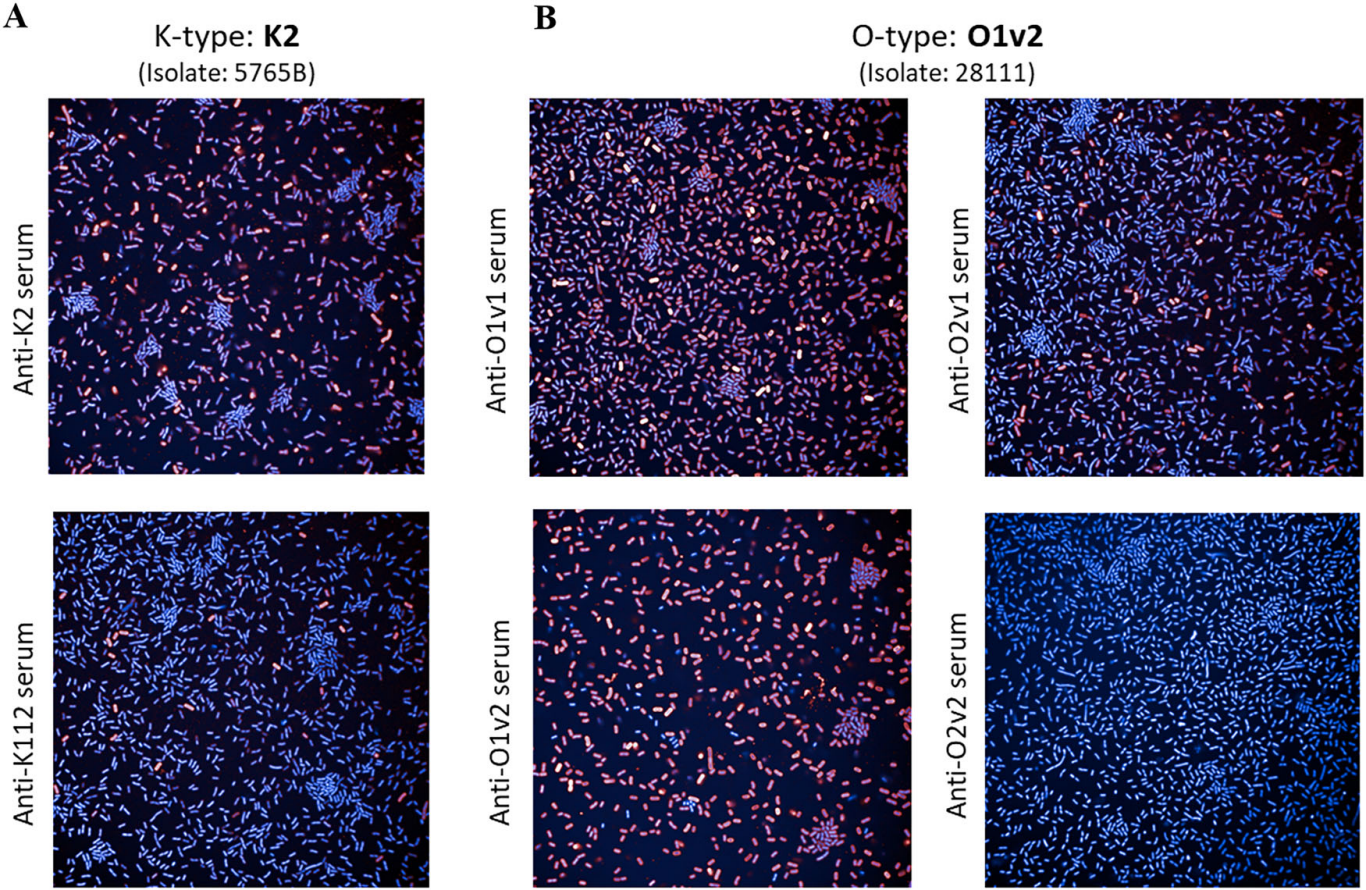
Bacterial imaging analysis. Binding of *K. pneumoniae* strains by homologous and heterologous anti -KAg and anti-OAg sera measured by High-content imaging. Strain 5765B bound by anti-K2 (homologous) and anti-KL112 (heterologous) sera (**A**); Strain 28111 bound by anti-O1v1 (homologous) and anti-O1v2, O2v1, and O2v2 (heterologous) sera (**B**). Red cells are bound by antibodies while the blue ones are not.

The cross-reactivity observed was additionally verified by high-content imaging, visualizing the binding of antibodies to individual bacterial cells (Fig. 8), and then we quantified the proportion of bacteria binding the antibodies. The data generated by high-content imaging duplicated the FC data for all the heterologous strains that were tested (Supplementary Fig. 7).

Cross-binding and cross-functionality were also investigated for anti-OAg antibodies for isolates sharing Gal-based OAg (71 in total). Anti-O1v1, O1v2, O2v1, and O2v2 antibodies were analyzed by FC and SBA against the panel of organisms with a Gal-based OAg (Supplementary Table 4).

**Fig. 9:**
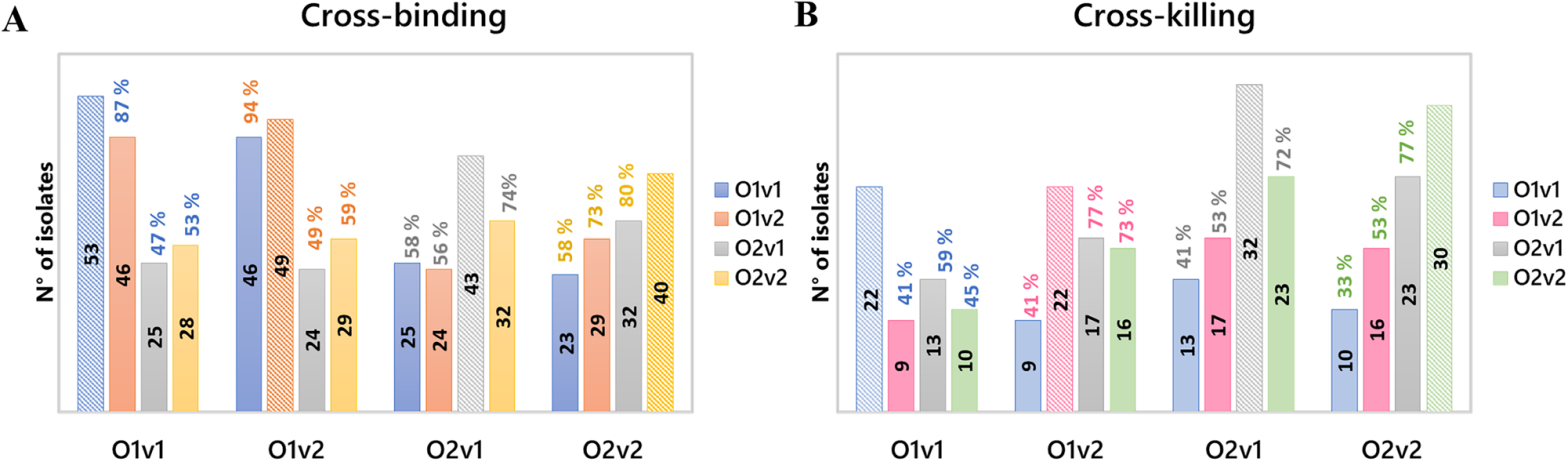
Bacterial binding and killing by homologous and heterologous OAg sera. Cross-binding (**A**) and cross-bactericidal activity (**B**) among Gal-based OAg. For each OAg type, the dotted bars represent the number of strains bound or killed by the homologous sera indicated on x axis, while full bars refer to number of the same strains bound or killed also by heterologous sera. Percentages are calculated as number of strains bound or killed from heterologous sera divided by total number of strains bound or killed from the homologous one.

Overall, we observed a high degree of cross-binding and cross-functionality. Indeed, among 53 isolates bound by anti-O1v1 antibodies, 46 (87%) were additionally bound by anti-O1v2 sera, 25 (47%) by anti-O2v1 and 28 (53%) by O2v2 antibodies (Fig. 9A). Results were confirmed for the isolates bound by O1v2 sera, witch again presented high cross-reactivity with O1v1 and O2 sera. For organisms displaying O2v1 or O2v2 OAg, > 50% cross-binding was verified for all heterologous sera. Comparable proportions were confirmed by SBA, specifically for O2 OAg (Fig. 9B). A lower proportion of cross-killing found for O1v1 and O1v2 may be explained by the greater resistance to killing observed in isolates displaying these OAg (Fig. 4). By interrogating MFI by FC for different sera against each organism, comparable binding was observed between O1v1 and O1v2, and O2v1 and O2v2, which translated into similar SBA titers (Supplementary Fig. 8).

## Discussion

Here, we determined the structural features of KAg and OAg displayed on a large panel of clinical *K. pneumoniae* isolates associated with neonatal sepsis in seven LMICs^30^. These analyses are important for confirming serotype distribution data obtained via genomic analysis; overall, our data demonstrated good matching with genomic prediction. We identified novel polysaccharide structures corresponding with KL102, KL105, KL108, KL112, KL116, KL117, KL122, KL128, and KL136, for which HPAEC-PAD analysis confirmed distinctive sugar composition compared to known structures. Here, K-types were assigned based on genomic analysis, while more in-depth structural characterization is ongoing to resolve their composition. Here, structural characteristics of KAg and OAg, identified as key virulence factors for *K. pneumoniae*^11^, were investigated with particular attention to sugar length and amount. We found that KAg is much longer than OAg and, in the growth conditions tested, generally expressed in higher amounts. This observation could result in masking of the sub-capsular OAg by the capsular polysaccharide, highlighting one of the key challenges for the development of an OAg-based vaccine against *K. pneumoniae*.

Many studies have shown the potential of anti-KAg antibodies to protect against *K. pneumoniae* in animal models^11^, while recent studies have highlighted some doubts about the role of anti-OAg antibodies to protect against capsulated isolates. Wantuch et al. compared K2- and O1-EPA bioconjugates for their immunogenicity and functional activity in murine models. Their findings indicated that the presence of high quantities of KAg can inhibit the binding and bactericidal activity of OAg specific antibodies. This masking effect was particularly notable in heavily encapsulated and hypervirulent variants. Correspondingly, in a murine model of bacteremia, mice immunized with O1-EPA bioconjugate had 100% mortality when challenged with a hypervirulent variant; in contrast, 100% of mice immunized with K2-EPA survived. When challenged with an encapsulated strain, the K2-EPA bioconjugate demonstrated a modest protection, which was statistically significant when compared to O1-EPA-immunized mice^20^. In a recent study, Hwang *et al*. found that the OAg was highly immunogenic in patients with *K. pneumoniae* bloodstream infection, with OAg antibody responses in patients significantly higher than antibody levels detected in healthy controls. However, physiological amounts of capsule produced by both hyper encapsulated and non-hyper encapsulated *K. pneumoniae* significantly inhibited OAg antibodies binding and functionality. This association was more evident with hypervirulent and hyper mucoid isolates and was strain dependent^21^. It was also reported that binding and phagocytic killing of OAg specific monoclonal antibodies to encapsulated strains belonging to different K-antigen serotypes was significantly reduced compared to corresponding non encapsulated mutants^19^. Also in this case, the extent of such reduction was serotype dependent, and the K2 antigen acted as the strongest penetration barrier, compared to other K-types.

The studies published to date to provide insights into the role of *K. pneumoniae* polysaccharides have been limited to a small number of serotypes (very often K2 and O1/O2) and a restricted set of specific strains. Results with monoclonal antibodies are affected by the specific epitope they recognize, its location on the bacterial surface, and their subclass^19^.

To further elucidate the role of KAg and OAg, sera from 7 common K-types and 6 O-types, reported in LMICs, were tested by FC and SBA against an extensive panel of strains collected from various regions. SBA assay represents a gold standard for many Gram-negative bacteria, like *Neisseria meningitidis*, *Shigella*, and *Salmonella*^31,32^, and has been already proposed to check functionality of sera against *K. pneumoniae*^33–36^. Furthermore, compared to OPK, we expect that this will be a more rapid mechanism of action of antibodies transferred from the mother to the neonate with no involvement of immune cells. For each K serotype, few isolates were tested to fix the percentage of exogenous complement to be used. Such amount was selected as the highest concentration not causing any aspecific killing for any of the isolates tested. This percentage of exogenous complement was then used for all strains sharing the same K type, to ensure comparability of results within isolates displaying the same KAg. Percentages of complement relatively high, between 20 and 50%, were used (Supplementary Table 5), indicating high resistance of Kp strains to complement. Interestingly, binding of anti-OAg antibodies was confirmed for all isolates independently from KAg characteristics. Also, a similar number of strains were killed by anti-OAg and anti-KAg antibodies. The panel of strains was tested somewhat biased, with 20 isolates (28%) belonging to an outbreak cluster of ST37 K15/O4 at the BARNARDS site in Ethiopia^1^, which were all successfully killed by both O4 and K15 antibodies. Excluding these ST37-K15/O4 strains, the fraction of other strains killed by OAg and KAg antibodies remained similar. In contrast to what was expected, no clear association was found between anti-OAg bactericidal activity and length or amount of the KAg. In these data, SBA appeared to be more strain specific: there must be some other feature of the strain that is impacting SBA, which is not captured by the specific KAg/OAg structural metrics measured here. Though it should be noted that strains were grown for structural characterization under different conditions than those used for SBA, to reach higher sugar expression levels. However, on a panel of 42 isolates, FC analysis was performed in parallel for the two conditions and found comparable results (Supplementary Fig. 9), suggesting that different growth conditions did not have major impacts on structural features.

It is difficult to predict if in vitro killing will translate to protection, and additional studies need to be performed toward the identification of a correlate of protection for *K. pneuomoniae*. A case-control study, to assess if serotype-specific anti-polysaccharide antibody responses and/or functionality are associated with a reduced risk of disease among neonates will be of great importance for *K. pneumoniae*, to help vaccine design and accelerate its development. This was recently successfully performed for Group B Streptococcus where capsular specific IgG concentrations were associated with a reduced risk of invasive disease among newborns^37^. Importantly, we have now developed in vitro assays (ELISA and SBA) that can support these kinds of studies.

Challenge experiments in animal models will be also useful to further understand the effectiveness of anti-KAg and anti-OAg antibodies, by working with a reasonably high number of isolates and more deeply investigating the characteristics of the strains in in vivo conditions.

There are challenges in the development of a vaccine against *K. pneumoniae*, especially related to the large diversity of capsular polysaccharides. Certain levels of cross-reactivity have been seen both by FC and SBA through our experiments. It is not clear at the moment if the levels of cross-reactivity found can be enough for cross-protection and simplification of vaccine design. Work is ongoing to explain cross-binding observed based on known primary sugar structures of the different KAg. Alternatively, the OAg represents an attractive target for vaccine development, due to its limited structural diversity^18,38,39^. Our data confirm significant cross-reactivity among structurally similar OAg types and subtypes, which could contribute to additional vaccine design simplification^21,35^. Notably, though, the most common antigens globally (O1)^1^ showed the lowest rate of OAg killing (Fig. 4).

Overall, our work provides a comprehensive set of data, through chemical-physical and immune characterization of a very large number of clinical isolates, which contributes to the design of a vaccine to prevent neonatal sepsis caused by *K. pneumoniae*. Our data suggest that both OAg and KAg can be included in a vaccine against *K. pneumoniae*, since KAg does not block antibodies binding to OAg and both anti-KAg and anti-OAg antibodies can mediate killing. Data on the global prevalence of K- and O-serotypes will be needed to identify optimal vaccine composition, and resistance to killing observed for certain serotypes can guide as well. Additional data will need to be generated to verify no negative immuno-interference for the final combination proposed and ability of the vaccine to protect in in vivo models.

## Materials and methods

### *K. pneuomoniae* strains and sequences

A panel of 150 isolates collected and sequenced in the BARNARDS study^1^ was shared by the Statens Serum Institute (SSI). Genomes were analyzed using Kaptive to identify the K and O loci and predicted serotypes^15^, and Kleborate v2 to identify the multi-locus sequence types^40^.

### Bacteria growth, KAg and OAg extraction, and isolation

All the *Klebsiella pneumoniae* isolates were inoculated overnight in LB medium (30 °C, 180 rpm) and then diluted at OD_600_ = 0.1 in 5 mL of M63 complete medium and incubated for 24 h at 37 °C, 180 rpm. Total saccharide extraction was achieved by adding AcOH to the culture broth to a final concentration of 2% v/v. After incubation for 2 h at 100 °C in a preheated thermoblock heater (Stuart SBH130D), the sample was centrifuged (4000 rpm, 10 min, 4 °C) and the supernatant was collected and filtered (Millipore membranes 0.22 µm)^24^. Finally, the absence of any bacterial contaminants was confirmed.

### Polysaccharide chemical characterization

Molecular weight estimation was performed by injecting 300 mL volume of AcOH supernatant on HPLC–SEC equipped with differential refractive index detector (dRI) using as eluent 0.1 M NaCl, 0.1 M NaH_2_PO_4_ pH 7.2 buffer. KAg MW were measured using TSK gel 6000PW (30 cm × 7.5 mm) column connected in series with a TSK gel 3000 PW_XL_ column (30 cm × 7.8 mm) and a TSK gel PW_XL_ guard column (4.0 cm × 6.0 mm) with a flow rate of 1 mL/min; pullulan standards were used to build a MW calibration curve (2000, 1000, 400, 200, 100, 50 kDa). OAg MW were measured using TSK gel 3000 PW_XL_ column (30 cm × 7.8 mm) connected in series with a TSK gel PW_XL_ guard column (4.0 cm × 6.0 mm) with a flow rate of 0.5 mL/min; pullulans standards were used to build a MW calibration curve (200, 100, 50, 22, 10, 6 kDa).

KAg and OAg polysaccharides separation before sugar composition analysis by HPAEC-PAD was achieved by HPLC–SEC by injecting a 300 mL volume of AcOH supernatant on TSK gel 3000 PW_XL_ column (30 cm × 7.8 mm) connected in series with a TSK gel PW_XL_ guard column (4.0 cm × 6.0 mm) and using as eluent 0.1 M NaCl, 0.1 M NaH_2_PO_4_ pH 7.2 buffer. The two polysaccharides were separately collected based on the retention times of the respective peaks using a GE Akta Frac-920 Fraction Collector.

Polysaccharide composition and amount were measured by HPAEC-PAD. KAg and OAg fractions pooled separately from HPLC-SEC were diluted to a final volume of 450 μL to have each sugar monomer in the range 0.5–10 μg/mL and hydrolyzed in 2 M trifluoroacetic acid (TFA) by using a Microwave Reaction System (Multiwave PRO, equipped with 4 × 20 MGC rotor, Anton Paar) reaching 700 W in 10 minutes with a linear power ramp and then maintaining 120 °C for 30 min with an irradiation power of 700 W. HPAEC-PAD analysis was performed as previously reported^24^.

### Synthesis of glycoconjugates

KAg and OAg were purified according to the purification protocol previously described^24^. Purified KAg was sonicated to reduce the molecular size by using a Sonics Vibra cell (BioClass, Pistoia, Italy) instrument for 30 min at 130 W, and size reduction was measured by HPLC-SEC analysis with pullulans standard (2000, 1000, 400, 200, 100, 50 kDa) as described above. All samples were lyophilized before re-solubilization in DABCO 100 mM pH 9 for following random conjugation to CRM_197_ carrier protein as recently described^27^. Conjugate formation was verified by HPLC-SEC analysis in comparison to free protein, and the conjugates were purified by ultrafiltration (100 kDa cut-off of the device).

### Generation of hyperimmune sera in rabbits

GSK is committed to the Replacement, Reduction, and Refinement of animal studies (3Rs). Non-animal models and alternative technologies are part of our strategy and employed where possible. When animals are required, the application of robust study design principles and peer review minimizes animal use, reduces harm, and improves benefits in studies. Animal studies were performed in Charles River Laboratories (France) in compliance with the relevant legislation (European Directive 2010/63/UE) and the institutional animal welfare of GSK.

Two or three New Zealand white rabbits per group received two intramuscular injections (500 mL volume) of 25 μg of KAg or OAg conjugated to CRM_197_ at day 0 and 28, and sera were collected at day 42. Anti-specific KAg and OAg IgG were measured by ELISA on single rabbits to confirm generation of antigen specific IgG (Supplementary Fig. 4). Pre-immune sera were also collected and tested via ELISA on the same coating antigens: IgG levels were undetectable for all antigens (data not shown). Sera from each immunization group were then pooled in equal volume from single animals to generate standard sera against each KAg/OAg then used for binding and bactericidal assays.

ELISA plates were coated with KAg (15 μg/mL) or OAg-HSA conjugates (obtained as the CRM_197_ conjugates described above) (1 mg/mL) in PBS, blocked with PBS milk 5%, and incubated with the sera diluted 1:100, 1:4000, and 1:160,000 in PBS milk 5%. Bound antibodies were then detected using an enzyme-labeled secondary antibody (anti-rabbit IgG-alkaline phosphatase) in PBS-Tween 0.05% 0.1% bovine serum albumin (BSA). The presence of immunoreacting anti-polysaccharide IgG was detected by the addition of substrate solution, with the formation of yellow color detected by measuring absorbance at 405 nm, subtracted by the absorbance at 490 nm. Results were expressed in ELISA units (EU/mL) which are equal the reciprocal of the dilution of the reference serum that yields an OD of 1 in the assay.

### Sera binding to *K. pneumoniae* isolates

*Klebsiella pneumoniae* cultures, grown under the same conditions used for KAg and OAg isolation, were harvested by centrifugation and washed twice in PBS. Bacterial pellets were incubated with standard pooled sera from rabbits immunized with KAg- and OAg-CRM_197_ conjugates diluted 1:500 and 1:1000, respectively for 1 h at RT. Following PBS washes, bacteria were incubated with Goat anti-Rabbit IgG Secondary Antibody, Alexa Fluor™ 488 (Invitrogen) diluted 1:1000 for 30 min at RT. Finally, bacteria were fixed in 4% formaldehyde (BD Cytofix, Bioscience, USA) for 30 min at 4 °C and resuspended in PBS. All washing steps and antibodies dilutions were performed using 1% (w/v) BSA in PBS. Each strain was also incubated with the Secondary Antibody only, as negative control. We considered positive by FC those samples for which MFI increase respect to negative control was > 4. 42 out of 150 strains were grown under the same conditions of L-SBA. Washing steps and staining with antibodies were performed as just described. For determination of bacteria binding with anti-KAg and anti-OAg antibodies a flow cytometer (BD Accuri C6 Plus Cytometer, BD Biosciences, San Jose, CA) equipped with a blue laser exciting at a wavelength of 488 nm was used. Before each experiment, the calibration of the flow cytometer was assessed with calibration beads (BDTM CS&T RUO Beads; BD Biosciences). Data acquisition was set at 10,000 events per sample, a flow rate of 66 µL /min, and a threshold at 5000 on FSC-Height signal. Data were collected using C6 Plus software, analyzed using FlowJo software (FlowJo, Ashland, Oregon) and reported as mean fluorescence intensity (Geometric Mean (FL1-A :: FITC-A)).

### Statistical Analysis

Statistical analysis and plots were obtained using JMP 15.2.0 and JMP 17.2.0 (SAS Institute Inc.).

### L-SBA

SBA conditions were established for each strain, starting from complement sensitivity tests. The same % of Baby Rabbit Complement (BRC), identified as the highest % not causing any aspecific killing was then used for all strains sharing same K type (50% for K2 and KL112, 30% for K5, K30, K 34, K39, K62, KL102, KL105 and KL128, 25% for K15, K18, K24 and K28, 20% for K25 and K64, respectively). Same conditions were used to test SBA of matching anti-OAg antibodies. Supplementary Table 5 reports results from experiments performed with selected strains with lower % of BRC respect to those selected for the analysis.

Standard hyperimmune sera were heat inactivated (56°C for 30 min) and tested against different K isolates in SBA based on luminescent readout adapting a method previously described^28,29^. Briefly, different dilutions of test sera were incubated with bacteria in 96-well round bottom sterile plates (Corning, Glendale, AZ, USA)–the SBA plate- in the presence of BRC. The HI sera were serially diluted in LB in the SBA plate (25 µL/well) starting from 1:20 dilution (final dilution) followed by 3-fold dilution steps up to 7 dilution points plus one control well with no sera, which represented control for non-specific complement killing as well as a sample diluted infinite-fold. Log-phase cultures for the assay were prepared as follows: frozen −80 °C bacterial working aliquots in 20% glycerol stocks were grown overnight (16–18 h) at 37 °C in M63 medium^24^, stirring at 180 rpm and then diluted in fresh LB medium in order to have an optical density at 600 nm (OD600) of 0.05 and incubated at 37 °C with 180 rpm agitation in an orbital shake, until reaching OD600 of 0.22 ± 0.03 and afterwards diluted to approximately 1 × 10^6^ Colony Forming Unit (CFU)/mL in LB. An adequate volume of reaction mixture containing the target bacterial cells (10 µL/well) and BRC as an external source of complement in LB was prepared; 75 µL/well of the reaction mixture were added to each well of the SBA plate containing HI serum dilutions (final reaction volume 100 µL), mixed and incubated for 3 h at 37 °C. At the end of the incubation, the SBA plate was centrifuged at room temperature for 10 min at 4000 × *g*. The supernatant was discarded to remove ATP derived from dead bacteria and SBA reagents. The remaining bacterial pellets were resuspended in PBS, transferred to a white 96-well plate (Greiner Bio-One, Roma, Italy) and mixed 1:1 v:v with BacTiter-Glo Reagent (Promega, Southampton, UK). The reaction was incubated for 5 min at room temperature on an orbital shaker at 600 rpm, and the luminescence signal was measured by a luminometer (Synergy HT, Biotek, Swindon, UK). A negative pre-immune control was added on each plate as well as a hyperimmune serum containing antibodies against an unrelated KAg, and no killing was verified.

In L-SBA the level of luminescence detected is directly proportional to the number of living bacteria present in the wells, which is inversely proportional to the level of functional antibodies present in the serum. A 4-parameter non-linear regression was applied to the raw luminescence (no normalization of data was applied) obtained for all the sera dilutions tested for each serum; an arbitrary serum dilution of 10^15^ was assigned to the well containing no sera. Fitting was performed by weighting the data for the inverse of luminescence^28,29^. To validate the dilution series, the highest luminescence detected in the dilution series at T180 had to be at least 0.7-fold the luminescence detected in the control well with no sera added. Results of the assay were expressed as the IC50, the reciprocal serum dilution that resulted in a 50% reduction of luminescence and thus corresponding to 50% growth inhibition of the bacteria present in the assay. GraphPad Prism software (GraphPad Software, La Jolla, CA, USA) was used for curve fitting and IC50 determination. A titer equal to half of the first dilution of sera tested (10) was assigned to not bactericidal sera.

### High content imaging (HCI) of bacteria

To evaluate bacterial population phenotypes, isolates were evaluated by HCI using the OPERA Phenix (PerkinElmer) and a protocol adapted from earlier work^41^. Briefly, 50 μL of inoculate (1/100 overnight liquid culture dilution) was added to each well of the Cell Carrier Ultra plate (Perkin Elmer), covered with an aerobic plate seal (Thermo Fischer) and incubated for 2.5 h at 37 °C 180 rpm. Excess inoculum was removed, and the cells were fixed using 50 μL of 4% PFA for 10–15 min before removing the excess. Each well was then washed once with PBS. PBS was then aspirated off each well, and 50 μL of 1 μg/mL polyclonal sera in 1% BSA solution was added to each well and left to incubate for 2 h at room temperature. The polyclonal sera solution was then removed and replaced with 50 μL of AF647 conjugated Ab (Mouse anti-Rabbit IgG, Thermo Fisher) (1/1000, 1 μg/mL) and DAPI (Sigma) (1/100, 1 μg/mL) diluted in 1% BSA solution and incubated for 20 min in the dark at room temperature. The excess was then removed, and 50 μL of PBS was then added. The plate was covered with a foil PCR plate seal (Thermo Fisher). To image, the 63x water immersion objective on the OPERA Phenix (Perkin Elmer) and the Alexa Fluor 647 and DAPI channels were utilized. Fluorescent intensity less than 600 was excluded based on optimization studies. 10 fields per well with 5–6z stacks approximately ~0.5 μm apart were used. All conditions were completed in triplicate biological and technical repeats. Controls were treated with only 1% BSA. Single stain controls were also used.

Image analysis was performed using Harmony (v4.9) based on Acapella software (v5.0.0.122842). The full analysis pipelines are included in Supplementary Fig. 8. All 6 z stacks for each field were concatenated into a single image using maximum projection. Optical correction was performed using flatfield and brightfield correction. Bacteria were identified and segmented using the SER ridge feature using the DAPI channel and filtered based on intensity to remove artefacts. Basic morphological features (area, length, width, roundness) were calculated for each object, while advanced features (symmetry, threshold compactness, axial, agglutination radial and profile measurements, stain intensity, binding percentage) were calculated for each of the AF647, DAPI, AF488 and/or AF546 channels for each object (experiment dependent). These features were used to filter artefacts based on size (excluding objects ≤1.5 μm^2^) and a linear classifier machine learning function (PhenoLogic, PerkinElmer). Objects were classified as either single cells, dividing cells, or artefacts. Pipeline training included over 100 objects manually classified into each group using a *Klebsiella* laboratory clinical reference isolate in the presence or absence of a range of compound concentrations and conditions. The approach was manually verified by visualizing the classification of wells from other plates imaged using the same experimental protocol.

### Reporting summary

Further information on research design is available in the Nature Portfolio Reporting Summary linked to this article.

## Supplementary information

Supplementary material

Reporting Summary
