# Supplementary material for: The characterization of Klebsiella pneumoniae associated with neonatal sepsis in low- and middle-income countries to inform vaccine design
Source: Commun Biol. 2025 Jun 9;8:898. doi: 10.1038/s42003-025-08258-7 (PMC12149307; doi:10.1038/s42003-025-08258-7)
Supplement: Supplementary file 1 — Supplementary material [file 42003_2025_8258_MOESM1_ESM.pdf]

The characterization of *Klebsiella pneumoniae* associated with neonatal sepsis in low- and middle-income countries to inform vaccine design

Francesca Nonne [1,*], Mariagrazia Molfetta [1,*], Gianina Florentina Belciug [1,*], Martina Carducci [1], Virginia Cianchi [2,3], Casey Zakroff [4], Salvatore Durante [4], Caroline Zellmer [5], Stephen Baker [6], Thomas D. Stanton [7,8], Kathryn E. Holt [7,9], Kelly Wyres [7,8], Neil Ravenscroft [10], Gianmarco Gasperini [4], Omar Rossi [1], Carlo Giannelli [1], Francesco Berlanda Scorza [1], Francesca Micoli [1,#]

[1] GSK Vaccines Institute for Global Health (GVGH), Siena, Italy;
[2] Department of Biomedical Sciences, Humanitas University, Milan, Italy;
[3] IRCCS Humanitas Research Hospital, Milan, Italy;
[4] GSK, Siena, Italy;
[5] The Department of Medicine, University of Cambridge, Cambridge, United Kingdom;
[6] A*STAR Infectious Diseases Labs (A*STAR IDL), Agency for Science, Technology and Research (A*STAR), Singapore;
[7] Department of Infectious Diseases, School of Translational Medicine, Monash University, Melbourne, Victoria, Australia;
[8] Centre to Impact AMR, Monash University, Clayton, Victoria, Australia;
[9] Department of Infection Biology, Faculty of Infectious and Tropical Diseases, London School of Hygiene and Tropical Medicine, London;
[10] Department of Chemistry, University of Cape Town, Cape Town, South Africa.

*These authors contributed equally
# Corresponding author: francesca.x.micoli@gsk.com

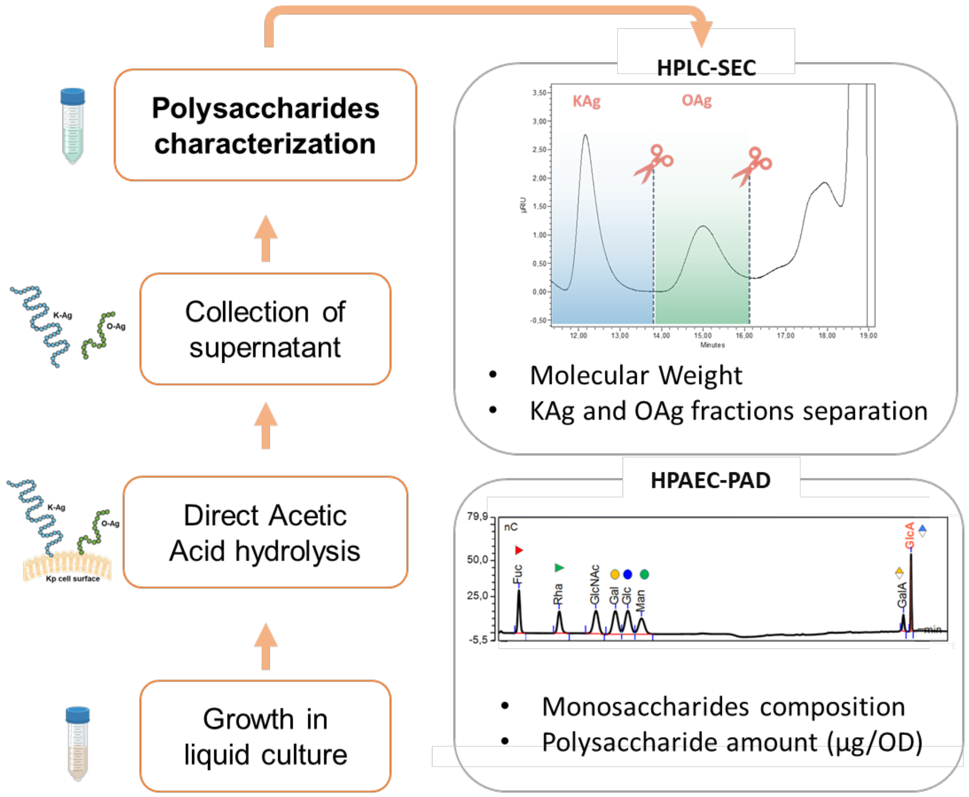

Supplementary Figure 1 | Scheme for KAg and OAg isolation from bacteria and chemical characterization.

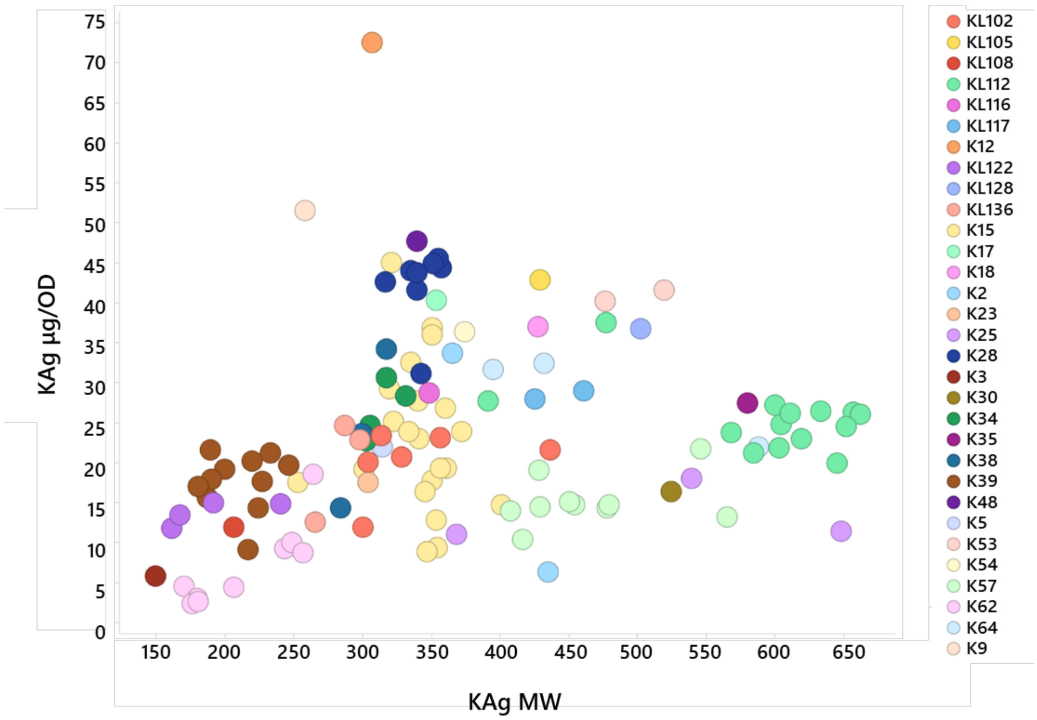

Supplementary Figure 2 | KAg structural features. Correlation between KAg amount (µg/OD) and size (MW).

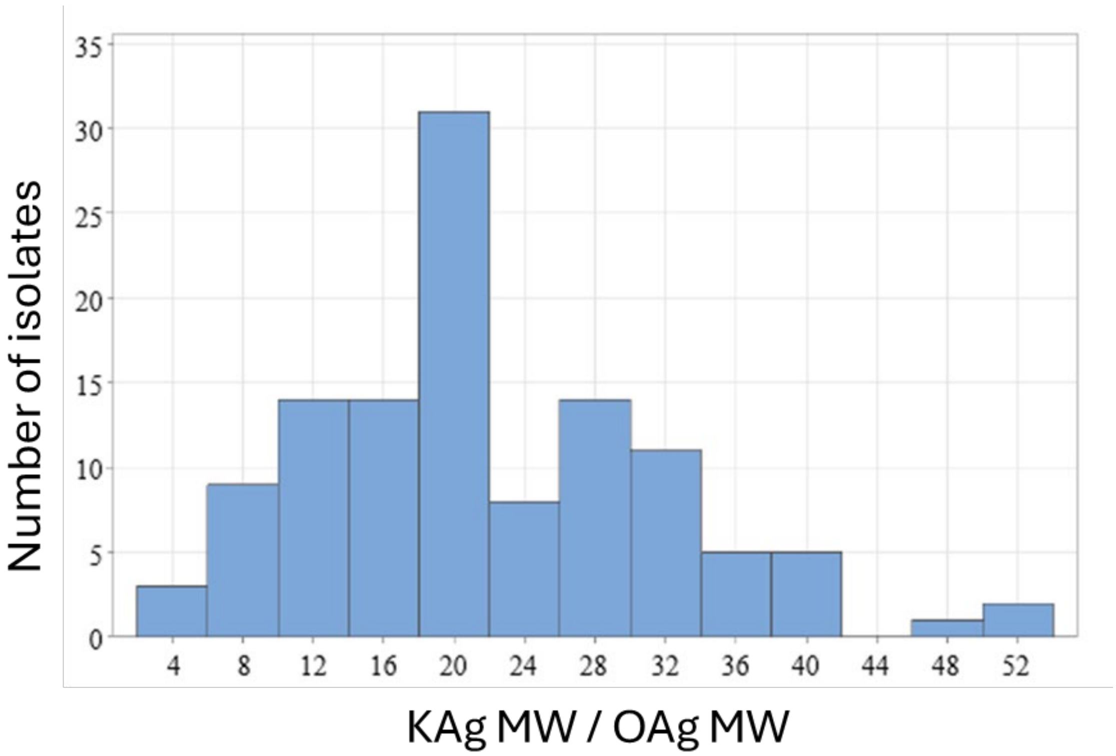

Supplementary Figure 3 | Number of isolates with a certain ratio of KAg MW/OAg MW.

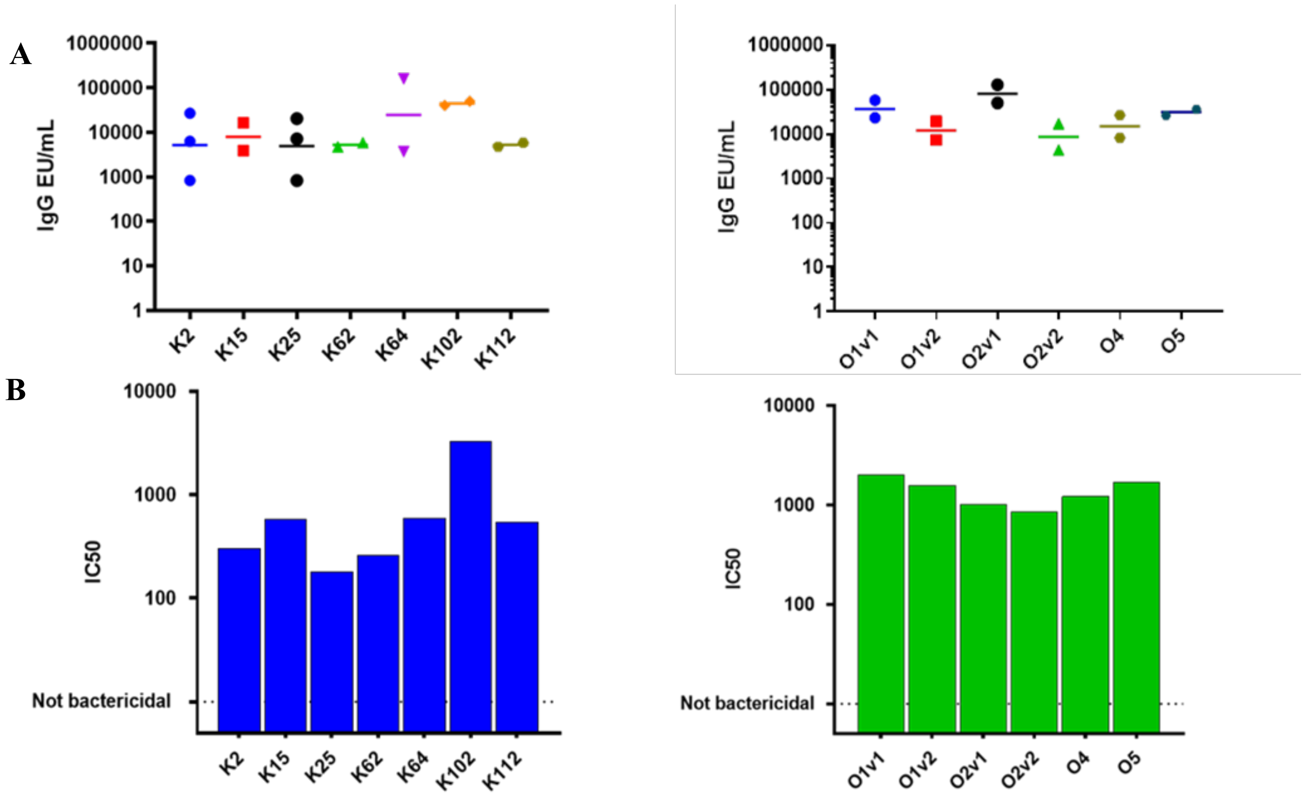

Supplementary Figure 4 | Evaluation of hyperimmune sera IgG and bactericidal activity via ELISA and SBA. Single sera from rabbits immunized with KAg and OAg glycoconjugates were analyzed by ELISA to check generation of specific antibodies against each serotype. Each dot corresponds to ELISA IgG/mL of single serum, while the line represents the geometric mean for each antigen (A). Pooled sera from each antigen were also tested for serum bactericidal activity (measured in terms of IC50) against a panel of selected strains expressing homologous KAg and OAg on their surface. Dotted line represents IC50 assigned to preimmune sera (B).

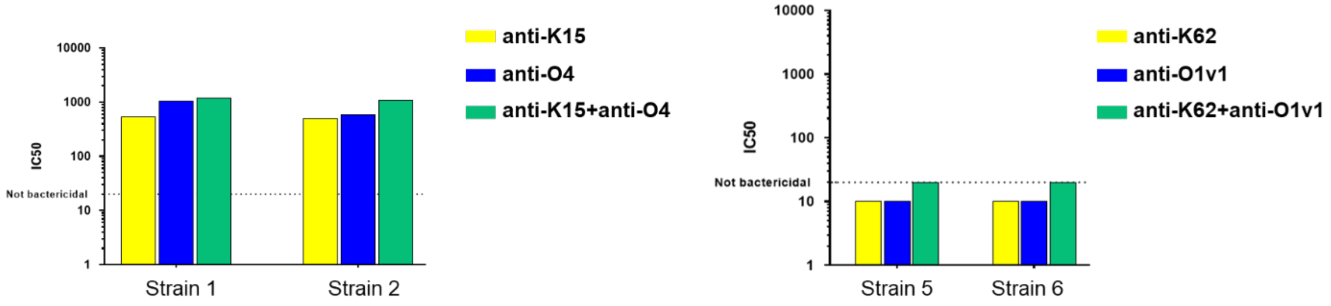

Supplementary Figure 5 | Evaluation of sera synergistic effect to kill selected bacteria. Specific anti-KAg and anti-OAg sera were pooled (1:1 in volume compared to 1:1 dilution with pre-immune sera when tested alone) and tested together in SBA against a small number of selected isolates. No synergistic effect from the combination of anti-KAg and anti-OAg antibodies was observed for either K15 or K62 strains.

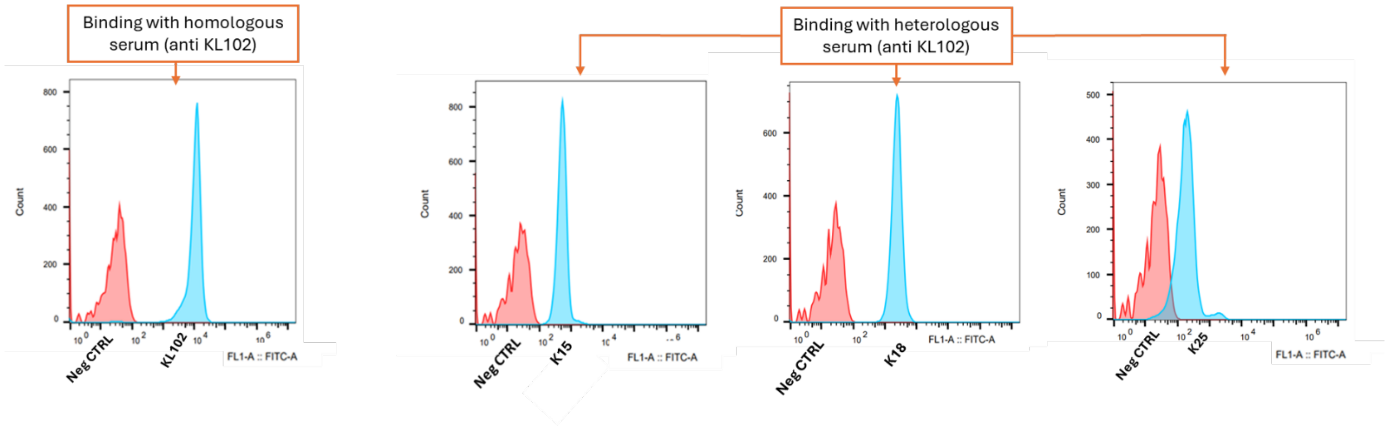

Supplementary Figure 6 | Representative FC graphs. The graphs show the binding of homologous (K102) and heterologous (K15, K18 and K25) sera to KL102 strain.

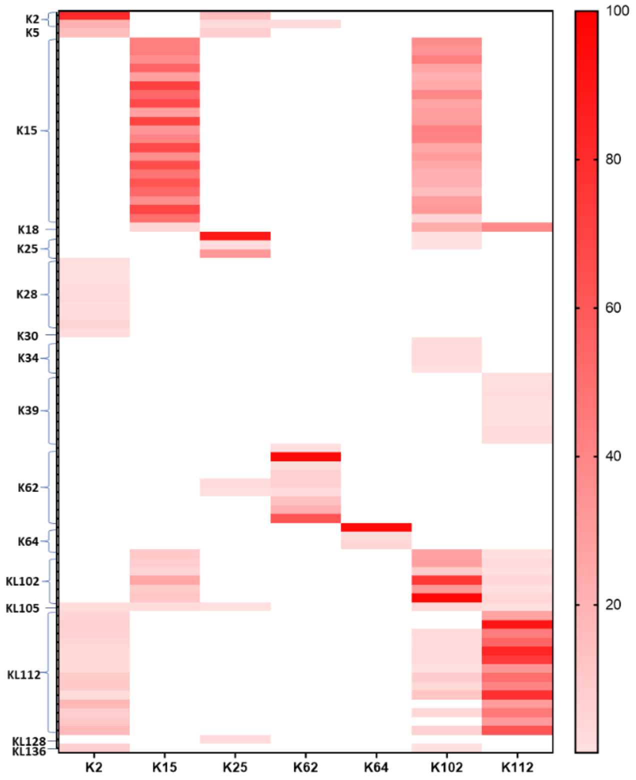

Supplementary Figure 7 | Binding measured by imaging of sera against heterologous strains. KAg specificity of the sera reported on the bottom of the graph tested against the panel of heterologous strains listed on the y-axis. Binding test was performed on isolates that gave positive signals in FC.

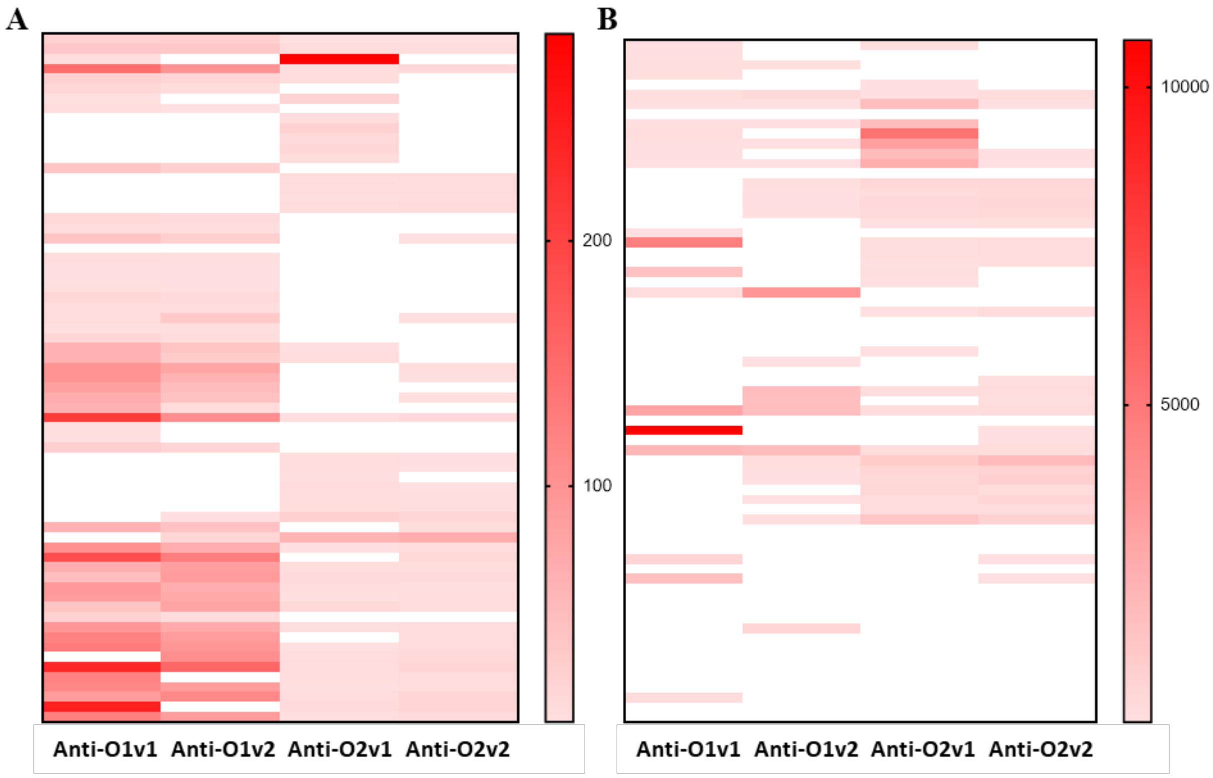

Supplementary Figure 8 | FC (A) and SBA (B) of sera listed on the bottom against panel of strains with Gal-based OAg. All isolates were tested with all sera by FC and SBA. FC: MFI fold increases respect to negative control. SBA: IC50 titers.

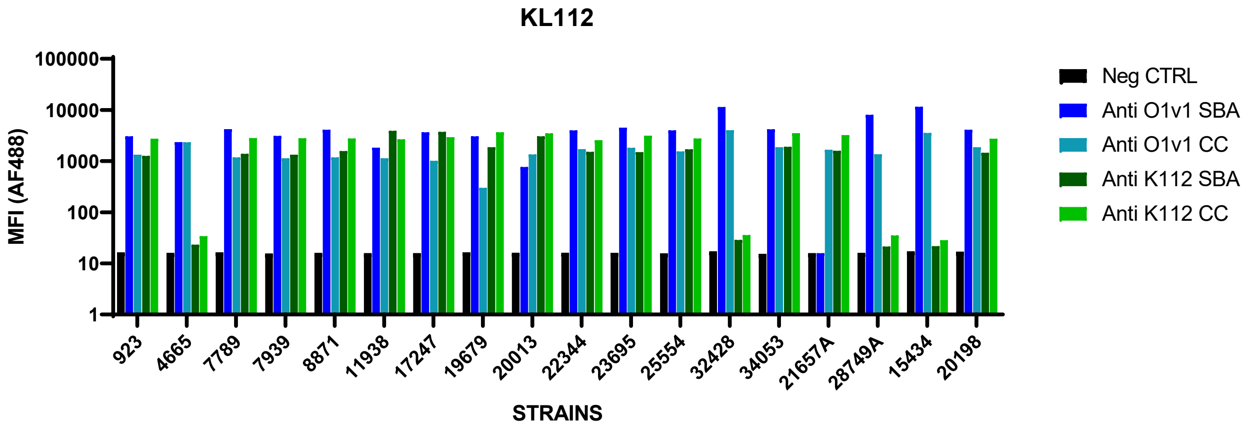

Supplementary Figure 9 | Evaluation of binding of hyperimmune sera against KL112 strains grown in different conditions. FC analyses performed in SBA and chemical characterization (CC) conditions, showing similar signals both for anti-KAg and anti-OAg antibodies independently from the growth conditions used. Results obtained with KL112 isolates are reported as an example; similar results were obtained for the other serotypes (data not shown).

Supplementary Table 1 | List of *K. pneumoniae* isolates characterized with main KAg and OAg genomic and structural characteristics and corresponding polysaccharide structures when available.

				KAg						OAg				
	**SSI Anon**	**ST**	**Origin**	**Genomic prediction**	**Chemical determination**	**Matching**	**MW (KDa)**	**µg/OD**	**K-Ag structure**	**Genomic prediction**	**Chemical determination**	**Matching**	**MW (KDa)**	**µg/OD**
**1**	5765B (Kp1)	ST39	South Africa	KL2	K2	Yes	435	6,4	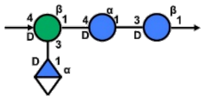	O1	Gal	Yes	36	8,3
**2**	4998 (Kp104)	ST14	Rwanda	QC fail	K2	N/A	365	33,7	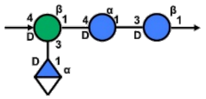	QC fail	Gal	N/A	20	9,5
**3**	5025 (Kp2)	ST13	Nigeria	KL3	K3	Yes	149	5,9	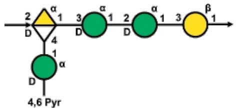	O1	Gal	Yes	34	9,1
**4**	5948 (Kp3)	ST14	Rwanda	KL2	K5	No	314	22,0	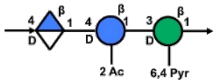	Untypeable	Gal	N/A	21	10,4
**5**	12771 (Kp4)	ST22	South Africa	KL9	K9	Yes	258	51,6	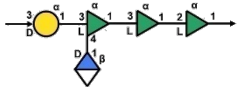	O2afg	Gal	Yes	11	10,8
**6**	28954 (Kp5)	ST163	South Africa	KL12	K12	Yes	307	72,6	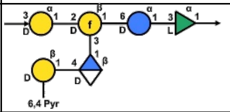	O1	Gal	Yes	20	13,0
**7**	1575 (Kp6)	ST37	Ethiopia	KL15	K15	Yes	354	9,4	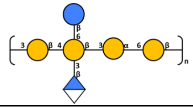	O4	O4	Yes	17	10,6
**8**	3890 (kp7)	ST37	Ethiopia	KL15	K15	Yes	361	19,3	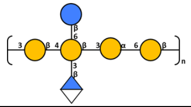	O4	O4	Yes	17	24,1

9	7648 (Kp8)	ST37	Ethiopia	KL15	K15	Yes	350	17,8	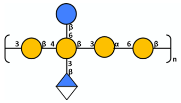	O4	O4	Yes	17	24,0
10	13647 (Kp9)	ST37	Ethiopia	KL15	K15	Yes	347	8,9	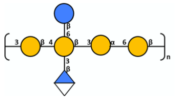	O4	O4	Yes	17	17,3
11	14427 (Kp10)	ST37	Ethiopia	KL15	K15	Yes	319	29,3	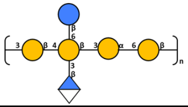	O4	O4	Yes	17	12,6
12	21120 (Kp11)	ST37	Ethiopia	KL15	K15	Yes	340	27,8	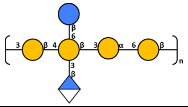	O4	O4	Yes	17	10,4
13	25827 (kp12)	ST37	Ethiopia	KL15	K15	Yes	353	12,8	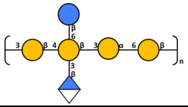	O4	O4	Yes	17	20,6
14	15383A (Kp13)	ST37	Ethiopia	No genome	K15	N/A	345	16,4	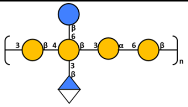	No genome	O4	N/A	17	23,5
15	15646B (Kp14)	ST37	Ethiopia	KL15	K15	Yes	360	26,8	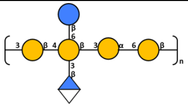	O4	O4	Yes	17	11,3
16	16960° (Kp15)	ST37	Ethiopia	KL15	K15	Yes	322	25,2	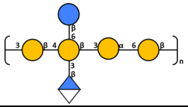	O4	O4	Yes	17	11,8
17	24767-2 (Kp16)	ST37	Ethiopia	KL15	K15	Yes	253	17,5	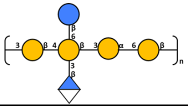	O4	O4	Yes	11	14,9
18	7008B (Kp17)	ST37	Ethiopia	KL15	No KAg detected	N/A	No KAg detected	No KAg detected	No KAg detected	Untypeable	O1v2	N/A	34	16,4
19	10988 (Kp18)	ST11	Nigeria	No genome	K15	N/A	401	14,8	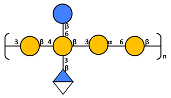	No genome	No OAg detected	N/A	No OAg detected	No OAg detected

20	1015 (Kp19)	ST37	Ethiopia	KL15	K15	Yes	372	23,9	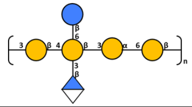	O4	O4	Yes	17	11,2
21	6560 (Kp20)	ST37	Ethiopia	KL15	K15	Yes	341	23,1	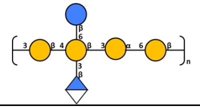	O4	O4	Yes	17	11,3
22	12200 (Kp21)	ST37	Ethiopia	KL15	K15	Yes	350	36,9	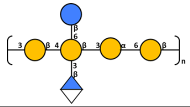	O4	O4	Yes	17	17,6
23	13447 (Kp22)	ST37	Ethiopia	KL15	K15	Yes	335	32,6	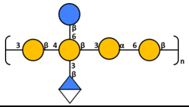	O4	O4	Yes	17	16,1
24	24847 (Kp23)	ST37	Ethiopia	KL15	K15	Yes	350	36,0	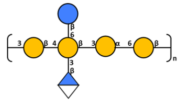	O4	O4	Yes	17	17,9
25	28111 (Kp24)	ST37	Ethiopia	KL15	No KAg detected	N/A	No KAg detected	No KAg detected	No KAg detected	O4	O1v2	No	34	17,2
26	28516 (Kp25)	ST37	Ethiopia	KL15	K15	Yes	333	23,9	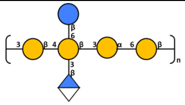	O4	O4	Yes	17	12,2
27	31381 (Kp26)	ST37	Ethiopia	KL15	K15	Yes	321	45,1	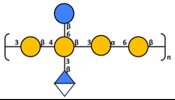	O4	O4	Yes	17	14,7
28	31132A (Kp27)	ST37	Ethiopia	KL15	K15	Yes	301	19,2	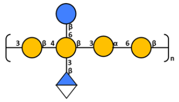	O4	O4	Yes	17	8,9
29	34658 (Kp90)	ST20	Pakistan	KL64	K15	No	356	19,4	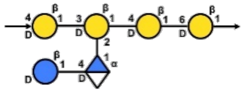	O1	O4	No	17	8,0
30	15567 (Kp28)	ST101	Bangladesh	KL17	K17	Yes	353	40,4	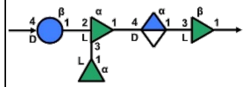	O1	Gal	Yes	32	6,2

31	3037 (Kp29)	ST967	India	KL18	K18	Yes	427	37,1	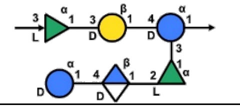	O1	Gal	Yes	22	15,7
32	298 (Kp30)	ST17	Bangladesh	KL23	K23	Yes	304	17,6	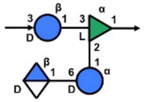	O1	Gal	Yes	23	14,7
33	23485 (Kp31)	ST45	Ethiopia	KL24	No KAg detected	N/A	No KAg detected	No KAg detected	No KAg detected	O2a	Gal	Yes	17	5,6
34	23415 (Kp32)	ST45	Ethiopia	KL24	No KAg detected	N/A	No KAg detected	No KAg detected	No KAg detected	O2a	Gal	Yes	34	17,5
35	27599 (Kp33)	ST607	Rwanda	KL25	K25	Yes	368	11,1	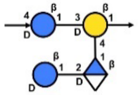	O1	Gal	Yes	33	8,0
36	29867 (Kp34)	ST17	Bangladesh	KL25	K25	Yes	648	11,4	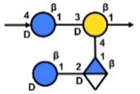	O5	Man	Yes	17	4,0
37	11610 (Kp117)	ST35	Ethiopia	No genome	K25	N/A	539	18,1	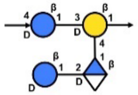	No genome	Gal	N/A	to low to be measured	1,5
38	1092 (Kp35)	ST20	Nigeria	KL28	K28	Yes	357	44,4	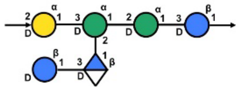	O1	Gal	Yes	21	12,5
39	12993 (Kp36)	ST442	Nigeria	KL28	K28	Yes	355	45,6	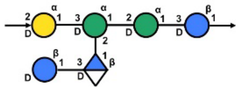	O2a	Gal	Yes	11	8,2
40	27152 (Kp37)	ST20	Nigeria	KL28	K28	Yes	351	44,9	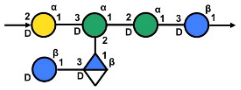	O1	Gal	Yes	21	11,5
41	1637 (Kp38)	ST442	Nigeria	KL28	K28	Yes	339	41,7	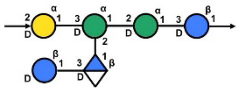	O2a	Gal	Yes	11	8,0

42	9098 (Kp39)	ST442	Nigeria	KL28	K28	Yes	342	31,2	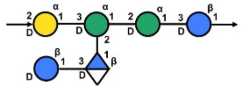	O2a	Gal	Yes	10	8,2
43	10319 (Kp40)	ST442	Nigeria	KL28	K28	Yes	335	44,1	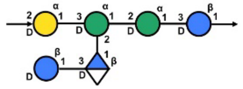	O2a	Gal	Yes	10	8,6
44	16698 (Kp41)	ST442	Nigeria	KL28	K28	Yes	339	43,8	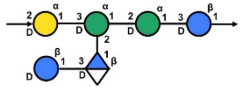	O2a	Gal	Yes	11	8,8
45	31931A (Kp42)	ST442	Nigeria	KL28	K28	Yes	316	42,6	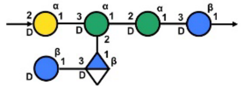	O2a	Gal	Yes	11	8,2
46	32482 (Kp43)	ST29	South Africa	KL30	K30	Yes	524	16,4	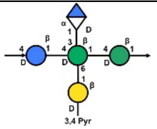	O1	Gal	Yes	36	7,4
47	1217 (Kp45)	ST76	Pakistan	KL31	?	No	215	23,5	Unidentified KAg	O3/O3a	Man	Yes	14	15,7
48	7703 (Kp46)	ST76	Nigeria	KL31	?	No	245	24,3	Unidentified KAg	O3/O3a	Man	Yes	13	16,9
49	25019 (Kp47)	ST76	Nigeria	KL31	?	No	224	25,6	Unidentified KAg	O3/O3a	Man	Yes	14	16,2
50	11078 (Kp48)	ST611	Pakistan	KL34	K34	Yes	302	22,8	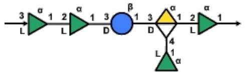	O2afg	Gal	Yes	11	9,9
51	17390 (Kp49)	ST611	Pakistan	KL34	K34	Yes	305	24,7	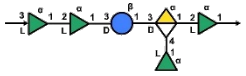	O2afg	Gal	Yes	11	10,1
52	15201 (Kp50)	ST611	Pakistan	KL34	K34	Yes	317	30,7	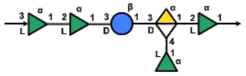	O2afg	Gal	Yes	11	10,3

53	29002 (Kp102)	ST87-1LV	Pakistan	Untypable	K34	N/A	331	28,4	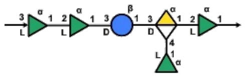	O2afg	Gal	Yes	11	10,7
54	22011 (Kp51)	ST12	Pakistan	KL35	K35	Yes	580	27,5	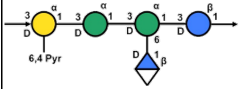	O3/O3a	Man	Yes	12	10,9
55	31154 (Kp52)	ST15	Bangladesh	KL38	K38	Yes	317	34,2	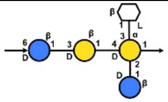	O3b	Man	Yes	10	7,8
56	16666 (Kp53)	ST292	Bangladesh	KL38	K38	Yes	299	23,7	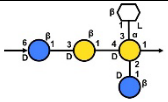	O3b	Man	Yes	11	6,6
57	17842 (Kp121)	ST395	Nigeria	No genome	K38	N/A	284	14,4	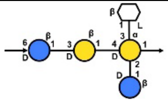	No genome	Man	N/A	11	4,6
58	651 (Kp54)	ST985	Ethiopia	KL39	K39	Yes	187	15,6	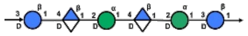	O1	Gal	Yes	20	23,3
59	8422 (Kp55)	ST985	Ethiopia	KL39	K39	Yes	189	21,7	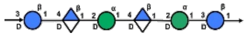	O1	Gal	Yes	20	13,4
60	8629 (Kp56)	ST985	Ethiopia	KL39	K39	Yes	224	14,4	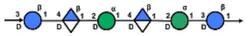	O1	Gal	Yes	20	12,0
61	11034 (Kp57)	ST985	Ethiopia	KL39	K39	Yes	246	19,7	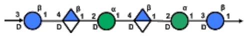	O1	Gal	Yes	21	11,7
62	26702 (Kp58)	ST985	Ethiopia	KL39	K39	Yes	227	17,7	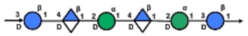	O1	Gal	Yes	21	11,7
63	17355B (Kp59)	ST985	Ethiopia	KL39	K39	Yes	199	19,2	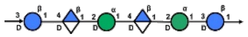	O1	Gal	Yes	21	15,2

64	2143A (Kp60)	ST985	Ethiopia	KL39	K39	Yes	190	18,0	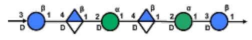	O1	Gal	Yes	21	14,1
65	10340 (Kp61)	ST985	Ethiopia	KL39	K39	Yes	180	17,1	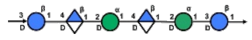	O1	Gal	Yes	21	13,6
66	4603 (Kp44)	ST391	Ethiopia	No genome	K39	N/A	233	21,2	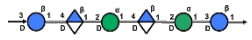	No genome	Gal	N/A	35	4,7
67	25004 (Kp73)	ST218	Ethiopia	KL57	K39	No	219	20,2	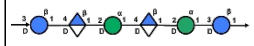	O2afg	Gal	Yes	21	12,5
68	29986 (Kp112)	ST35	Ethiopia	KL108	K39	No	216	9,1	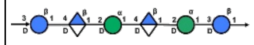	O1	Gal	Yes	21	7,9
69	16394 (Kp62)	ST15	Nigeria	KL48	K48	Yes	339	47,7	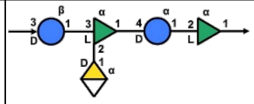	O1	Gal	Yes	20	12,5
70	3314 (Kp63)	ST464	Nigeria	KL53	?	No	467	30,1	Unidentified KAg	O1	Gal	Yes	22	12,4
71	12173 (Kp64)	ST464	Nigeria	KL53	No KAg detected	N/A	No KAg detected	No KAg detected	No KAg detected	O1	Gal	Yes	20	23,7
72	16514 (Kp65)	ST464	Nigeria	KL53	K53	Yes	519	41,6	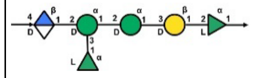	O1	Gal	Yes	21	15,4
73	7161 (Kp66)	ST464	Nigeria	KL53	K53	Yes	476	40,2	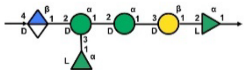	O1	Gal	Yes	20	14,0
74	24344 (Kp67)	ST170	Nigeria	KL54	K54	Yes	374	36,4	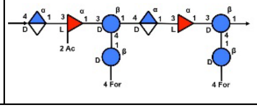	O1	Gal	Yes	20	12,0

**75**	29558A (Kp68)	ST711	Ethiopia	KL54	?	No	337	16,3	Unidentified KAg	O1	Gal	Yes	22	12,9
**76**	26169 (Kp69)	ST1035	Nigeria	KL57	K57	Yes	565	13,2	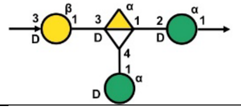	O1	Gal	Yes	22	22,0
**77**	1642 (Kp70)	ST218	Ethiopia	KL57	K57	Yes	428	19,1	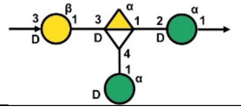	O2afg	Gal	Yes	12	9,1
**78**	4145 (Kp71)	ST218	Ethiopia	KL57	K57	Yes	454	14,8	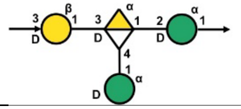	O2afg	Gal	Yes	11	8,2
**79**	18432 (Kp72)	ST218	Ethiopia	KL57	K57	Yes	478	14,4	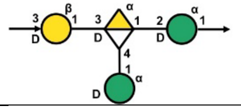	O2afg	Gal	Yes	12	8,3
**80**	17057B (Kp74)	ST218	Ethiopia	KL57	K57	Yes	429	14,5	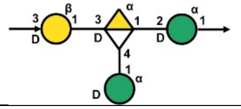	O2afg	Gal	Yes	12	9,0
**81**	20044B (Kp75)	ST218	Ethiopia	KL57	K57	Yes	450	15,1	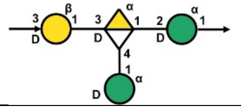	O2afg	Gal	Yes	12	9,1
**82**	24412A (Kp76)	ST218	Ethiopia	KL57	K57	Yes	407	14,0	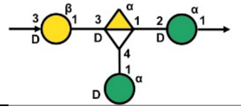	O2afg	Gal	Yes	12	8,6
**83**	8402 (Kp77)	ST218	Ethiopia	KL57	K57	Yes	479	14,8	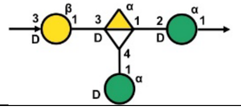	O2afg	Gal	Yes	12	8,7
**84**	7972B (Kp78)	ST218	Ethiopia	KL57	K57	Yes	416	10,4	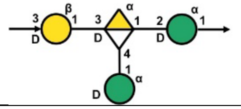	O2afg	Gal	Yes	12	7,6
**85**	24649 (Kp79)	ST592	Nigeria	KL57	K57	Yes	546	21,8	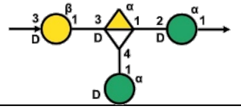	O3b	Man	Yes	11	8,9

86	17681-1 (Kp80)	ST348	South Africa	KL62	K62	Yes	243	9,3	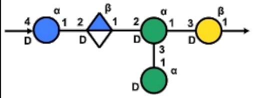	O1	Gal	Yes	19	13,2
87	17681-2 (Kp81)	ST348	South Africa	KL62	K62	Yes	248	10,1	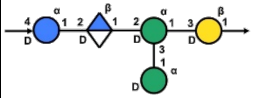	O1	Gal	Yes	19	13,3
88	20863 (Kp82)	ST2534	India	KL62	K62	Yes	256	8,8	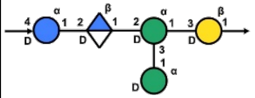	O1	Gal	Yes	36	8,8
89	21997 (Kp83)	ST348	South Africa	KL62	K62	Yes	264	18,6	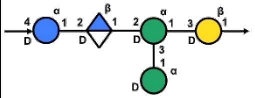	O1	Gal	Yes	31	15,2
90	8094 (Kp84)	ST39	Etiopia	KL62	K62	Yes	170	4,6	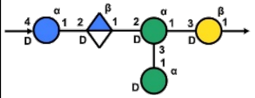	O1	Gal	Yes	41	11,2
91	8645 (Kp85)	ST39	Nigeria	KL62	K62	Yes	206	4,4	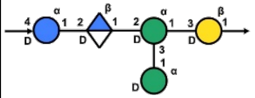	O1	Gal	Yes	43	9,2
92	31876 (Kp86)	ST48	Rwanda	KL62	K62	Yes	179	3,0	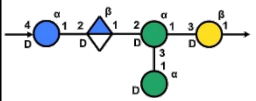	O1	Gal	Yes	36	7,2
93	8496A (Kp87)	ST530	Nigeria	KL62	K62	Yes	176	2,4	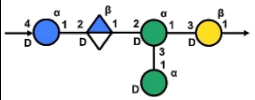	O1	Gal	Yes	37	9,6
94	9818 (Kp88)	ST48	Rwanda	KL62	K62	Yes	180	2,6	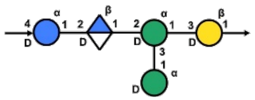	O1	Gal	Yes	33	7,5
95	29774 (Kp89)	ST14	Bangladesh	KL64	No KAg detected	N/A	No KAg detected	No KAg detected	No KAg detected	O1	Gal	Yes	30	15,3
96	11522 (Kp91)	ST2986	Rwanda	No genome	K64	N/A	395	31,7	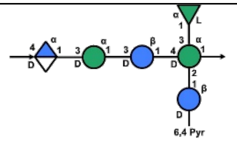	No genome	Gal	N/A	33	10,3

**97**	33058 (Kp92)	ST2986	Rwanda	KL64	K64	Yes	432	32,5	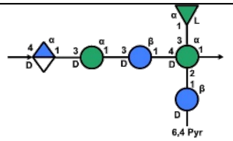	O1	Gal	Yes	33	10,1
**98**	18936 (Kp93)	ST14	Bangladesh	KL64	K64	Yes	588	22,0	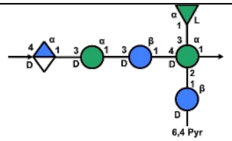	O1	Gal	Yes	31	7,7
**99**	8139 (Kp94)	ST307	Rwanda	KL102	K102	Yes	328	20,7	Unknown structure	O2afg	Gal	Yes	18	9,0
**100**	10004 (Kp95)	ST307	Rwanda	KL102	K102	Yes	304	20,1	Unknown structure	O2afg	Gal	Yes	18	9,2
**101**	12641B (Kp96)	ST307	Rwanda	KL102	K102	Yes	436	21,7	Unknown structure	O2afg	Gal	Yes	17	10,7
**102**	17814 (Kp97)	ST105	Bangladesh	KL102	K102	Yes	356	23,2	Unknown structure	O2afg	Gal	Yes	17	10,7
**103**	2051 (Kp98)	ST307	Rwanda	KL102	K102	Yes	313	23,4	Unknown structure	O2afg	Gal	Yes	18	9,4
**104**	4415 (Kp99)	ST307	Rwanda	KL102	K102	Yes	300	12,0	Unknown structure	O2afg	Gal	Yes	18	10,0

**105**	34831 (Kp103)	ST307	Nigeria	KL102	No KAg detected	N/A	No KAg detected	No KAg detected	No KAg detected	O2afg	Gal	Yes	12	20,7
**106**	17648 (Kp100)	ST11	Bangladesh	KL105	K105	Yes	429	42,9	Unknown structure	O2afg	Gal	Yes	11	11,1
**107**	471 (Kp105)	ST35	Ethiopia	KL108	No KAg detected	N/A	No KAg detected	No KAg detected	No KAg detected	O1	Gal	Yes	21	22,7
**108**	1775 (Kp106)	ST35	Ethiopia	KL108	No KAg detected	N/A	No KAg detected	No KAg detected	No KAg detected	O1	Gal	Yes	21	24,6
**109**	12024 (Kp107)	ST35	Ethiopia	KL108	No KAg detected	N/A	No KAg detected	No KAg detected	No KAg detected	O1	Gal	Yes	21	25,3
**110**	17740 (Kp108)	ST35	Ethiopia	KL108	No KAg detected	N/A	No KAg detected	No KAg detected	No KAg detected	O1	Gal	Yes	21	25,4
**111**	20384 (Kp109)	ST35	Ethiopia	KL108	No KAg detected	N/A	No KAg detected	No KAg detected	No KAg detected	O1	Gal	Yes	22	23,7
**112**	23406 (Kp110)	ST35	Ethiopia	KL108	No KAg detected	N/A	No KAg detected	No KAg detected	No KAg detected	O1	Gal	Yes	21	17,6
**113**	27411 (Kp111)	ST35	Ethiopia	KL108	No KAg detected	N/A	No KAg detected	No KAg detected	No KAg detected	O1	Gal	Yes	22	14,6
**114**	34310 (Kp113)	ST35	Ethiopia	KL108	No KAg detected	N/A	No KAg detected	No KAg detected	No KAg detected	O1	Gal	Yes	21	17,9
**115**	27749B (Kp114)	ST35	Ethiopia	KL108	No KAg detected	N/A	No KAg detected	No KAg detected	No KAg detected	O1	Gal	Yes	21	17,2

116	34715A (Kp115)	ST35	Ethiopia	KL108	No KAg detected	N/A	No KAg detected	No KAg detected	No KAg detected	O1	Gal	Yes	22	21,0
117	359 (Kp116)	ST35	Ethiopia	KL108	No KAg detected	N/A	No KAg detected	No KAg detected	No KAg detected	O1	Gal	Yes	21	21,7
118	20018 (Kp118)	ST35	Ethiopia	KL108	No KAg detected	N/A	No KAg detected	No KAg detected	No KAg detected	O1	Gal	Yes	21	21,8
119	14880A (Kp119)	ST35	Ethiopia	KL108	No KAg detected	N/A	No KAg detected	No KAg detected	No KAg detected	O1	Gal	Yes	21	21,6
120	27870B (Kp120)	ST35	Ethiopia	KL108	K108	Yes	206	11,9	Unknown structure	O1	Gal	Yes	21	10,8
121	923 (Kp122)	ST15	Pakistan	KL112	K112	Yes	600	27,2	Unknown structure	O1	Gal	Yes	21	14,0
122	4665 (Kp123)	ST15	Pakistan	KL112	No KAg detected	N/A	No KAg detected	No KAg detected	No KAg detected	O1	Gal	Yes	20	12,9
123	7789 (Kp124)	ST15	Pakistan	KL112	K112	Yes	568	23,8	Unknown structure	O1	Gal	Yes	20	11,9
124	7939 (Kp125)	ST15	Pakistan	KL112	K112	Yes	604	24,8	Unknown structure	O1	Gal	Yes	21	13,1

**125**	8871 (Kp126)	ST15	Pakistan	KL112	K112	Yes	603	21,9	Unknown structure	O1	Gal	Yes	21	12,0
**126**	11938 (Kp127)	ST15	Pakistan	KL112	K112	Yes	633	26,5	Unknown structure	O1	Gal	Yes	21	12,5
**127**	17247 (Kp128)	ST15	Pakistan	KL112	K112	Yes	611	26,2	Unknown structure	O1	Gal	Yes	22	12,3
**128**	19679 (Kp129)	ST15	India	KL112	K112	Yes	477	37,6	Unknown structure	O1	Gal	Yes	20	14,0
**129**	20013 (Kp130)	ST15	Pakistan	KL112	K112	Yes	391	27,8	Unknown structure	O1	Gal	Yes	20	11,5
**130**	22344 (Kp131)	ST15	Pakistan	KL112	K112	Yes	584	21,2	Unknown structure	O1	Gal	Yes	20	13,0
**131**	23695 (Kp132)	ST15	Pakistan	KL112	K112	Yes	619	23,0	Unknown structure	O1	Gal	Yes	20	11,0

132	25554 (Kp133)	ST15	Pakistan	KL112	K112	Yes	657	26,3	Unknown structure	O1	Gal	Yes	21	12,5
133	32428 (Kp134)	ST15	Pakistan	KL112	No KAg detected	N/A	No KAg detected	No KAg detected	No KAg detected	O1	Gal	Yes	22	13,4
134	34053 (Kp135)	ST15	Pakistan	KL112	K112	Yes	645	20,0	Unknown structure	O1	Gal	Yes	20	12,6
135	21657A (Kp136)	ST15	Pakistan	KL112	K112	Yes	662	26,1	Unknown structure	O1	Gal	Yes	21	13,3
136	28749A (Kp137)	ST17	Ethiopia	KL112	No KAg detected	N/A	No KAg detected	No KAg detected	No KAg detected	O2afg	Gal	Yes	21	27,1
137	15434 (Kp138)	ST15	Pakistan	KL112	No KAg detected	N/A	No KAg detected	No KAg detected	No KAg detected	O1	Gal	Yes	22	14,1
138	20198 (Kp139)	ST15	Pakistan	KL112	K112	Yes	652	24,6	Unknown structure	O1	Gal	Yes	21	12,4
139	25484 (Kp140)	ST39	Nigeria	KL116	No KAg detected	N/A	No KAg detected	No KAg detected	No KAg detected	O1	Gal	Yes	25	8,1
140	24745-2 (Kp141)	ST4410	Nigeria	KL116	K116	Yes	348	28,8	Unknown structure	O1	Gal	Yes	18	9,0

141	10405 (Kp142)	ST995	Pakistan	KL117	K117	Yes	461	29,0	Unknown structure	O1	Gal	Yes	19	10,1
142	4433 (Kp143)	ST995	Pakistan	KL117	K117	Yes	425	28,0	Unknown structure	O1	Gal	Yes	19	10,9
143	773 (Kp144)	ST17	Rwanda	KL122	K122	Yes	191	15,0	Unknown structure	O1	Gal	Yes	28	13,4
144	33090 (Kp145)	ST17	South Africa	KL122	K122	Yes	161	11,8	Unknown structure	O1	Gal	Yes	28	13,3
145	10235 (Kp146)	ST17	Rwanda	KL122	K122	Yes	167	13,5	Unknown structure	O2afg	Gal	Yes	28	13,0
146	12933 (Kp147)	ST17	Bangladesh	KL122	K122	Yes	240	14,9	Unknown structure	O2afg	Gal	Yes	12	8,2
147	30405 (Kp148)	ST147	Bangladesh	KL128	K128	Yes	502	36,8	Unknown structure	O3b	Man	Yes	10	13,1
148	6641 (Kp149)	ST70	Pakistan	KL136	K136	Yes	287	24,7	Unknown structure	O1	Gal	Yes	20	12,1
149	34473 (Kp150)	ST70	Rwanda	KL136	K136	Yes	298	22,9	Unknown structure	O1	Gal	Yes	19	12,9
150	16508 (Kp101)	ST70	Pakistan	KL136	K136	Yes	265	12,6	Unknown structure	O1	Gal	Yes	20	14,5

#For KL>100, structure determination is ongoing; matching was indicated based on the fact that sugar composition determined by HPAEC-PAD was different respect to known KAg structures and that all strains having same K-type assignment shared same sugar composition.

Supplementary Table 2 | List of glycoconjugates prepared for generation of hyperimmune sera in rabbits.

**Conjugates**	**Saccharide/protein molar ratio***
K2-CRM_{197}	0.53
K15-CRM_{197}	0.98
K25-CRM_{197}	0.56
K62-CRM_{197}	0.96
K64-CRM_{197}	0.98
K102-CRM_{197}	0.87
K112-CRM_{197}	0.62
O1v1-CRM_{197}	0.43
O1v2-CRM_{197}	0.47
O2v1-CRM_{197}	0.35
O2v2-CRM_{197}	0.39
O4-CRM_{197}	0.45
O5-CRM_{197}	0.55

*calculated by phenol sulfuric acid assay or HPAEC-PAD and micro BCA

Supplementary Table 3 | Binding by FC and SBA of selected KAg sera against a panel of Kp isolates sharing homologous or heterologous K-types.

		anti-K2		*anti-K15		anti-K25		anti-K62		anti-K64		anti-K102		anti-K112			
**Strain**	**KAg**	**MFI FC**	**SBA**	**MFI FC**	**SBA**	**MFI FC**	**SBA**	**MFI FC**	**SBA**	**MFI FC**	**SBA**	**MFI FC**	**SBA**	**MFI FC**	**SBA**	**neg CTRL MFI**	***neg CTRL K15 MFI**
**5765B**	K2	1462	303	31.7	57	77.9	37	28.7	10	21.5	10	21.2	10	19.3	10	16.4	24.4
**4998**	K2	4740	203	14.6	10	214	10	123	10	32.1	10	33.5	10	27.8	10	22.6	12.8
**5948**	K5	1496	25	23.9	10	94.1	10	35	22	33.6	10	21.1	82	19.3	65	16.5	21
**1575**	K15	21.6	10	2029	207	24.3	10	20.8	10	24.6	10	431	53	22.7	55	15.7	21.7
**3890**	K15	21.4	10	2186	247	23.7	10	20.3	10	24.1	10	395	21	22.5	10	15.9	25.1
**7648**	K15	21.2	10	2460	305	23.7	10	20.5	10	23.4	10	417	55	22.9	10	15.8	24.3
**13647**	K15	21.4	10	2178	489	24.1	10	21.1	10	23.8	10	432	25	23.3	10	15.9	23.7
**14427**	K15	22.4	10	2530	439	25.6	10	21	10	24.8	10	523	20	24.1	10	15.9	23.8
**21120**	K15	21.3	10	2439	210	24.3	10	20.5	10	24.5	10	452	21	24.2	10	15.9	25.7
**25827**	K15	20.9	65	2138	203	23.7	44	20.9	10	24.1	10	425	56	22.9	10	15.6	23.6
**15383A**	K15	18.7	10	2280	205	22.6	10	20.2	10	22.5	10	412	10	24.2	10	15.7	24.1
**15646B**	K15	18.7	23	2488	202	22.6	10	20.2	10	23.1	147	408	57	24.2	10	15.2	24
**16960A**	K15	18.4	10	2146	214	22.6	10	19.6	10	22.6	10	378	10	23.1	10	15.3	23.7
**24767-2**	K15	18.6	10	1978	206	24	10	20.4	10	24.4	10	448	24	24.4	10	15.3	23.6
**7008B**	K15	21.7	10	38.3	10	26.6	10	21.8	10	24.8	10	34.4	10	22.9	10	15.5	22.9
**10988**	K15	18.7	10	1338	214	20.8	10	20.2	10	20.9	10	121	10	21	10	15.4	22.4
**1015**	K15	19.9	10	1939	360	23.6	10	21.7	10	24	10	420	10	25.6	10	16	24.1
**6560**	K15	22.2	10	2352	352	27.2	10	23.9	10	27.2	10	485	36	27.3	10	17	24.5
**12200**	K15	19.5	10	2068	442	23.2	10	20.4	10	24.2	10	390	31	24.3	10	15.5	24.3
**13447**	K15	20.9	10	2027	586	24.7	10	22.4	10	24.8	10	447	53	25.8	10	15.9	26.9
**24847**	K15	17.9	10	2015	534	20.9	21	18.8	10	20.8	10	399	22	21.1	10	15.2	23.7
**28111**	K15	20.1	10	40.5	10	25.5	10	19.4	10	22.1	26	32.9	26	21.8	10	15.5	22.9
**28516**	K15	19.6	10	2199	228	21.2	10	19.4	10	21.6	10	336	44	19.9	10	15.3	23.5
**31381**	K15	19.7	10	2142	194	20.9	10	18.3	21	21.1	10	340	10	18.5	10	14.9	24.3
**31132A**	K15	19.3	10	2068	196	21.2	10	17.8	10	20.8	10	333	49	19.5	10	15.2	23.9
**34658**	K15	30.7	21	1466	537	32.1	10	29.3	10	31.3	10	1008	66	32	10	19.6	21
**3037**	K18	24.8	33	611	10	39.9	10	21.7	10	38.6	10	2239	10	110	10	14.9	19.6
**23485**	K24	21.4	22	47.7	142	22.3	75	19.5	63	24.2	20	27.3	10	26.6	60	16	22.6
**23415**	K24	25.9	10	36.7	10	28.1	10	23.1	10	26.7	10	32.8	10	22.5	10	16.1	20.3
**27599**	K25	20.8	10	29.2	10	1578	10	21.6	10	24.4	10	146	10	22.5	10	15.8	18.9
**29867**	K25	19.7	56	39	46	1491	181	20.5	10	23.9	10	176	10	22.1	10	15	22.7
**11610**	K25	38.8	10	19.1	10	2414	10	37.8	10	43.1	10	77.2	10	40.1	10	27.6	12.9
**1092**	K28	72.9	10	41.2	10	35.8	56	31.3	10	31.2	10	58.3	70	48	61	15.2	20.5
**12993**	K28	72.7	58	42.1	10	30.8	10	26.3	10	28	10	45.7	60	43	10	15.6	21.1
**27152**	K28	78.4	10	37.9	10	37.4	10	31.3	10	33.1	10	56	34	47.5	10	15.8	21.9
**1637**	K28	73.5	36	41.3	54	34.7	10	27.6	10	30.6	10	51.2	95	42.8	47	15.4	21.8
**9098**	K28	68.6	60	31.1	61	33.8	10	30	49	32.6	23	55.5	101	42.4	10	15.9	20.8
**10319**	K28	80.7	25	36.6	61	37.1	55	31.5	72	34.6	10	61.7	108	47.9	60	16.2	21.1
**16698**	K28	83	55	35.7	10	35.2	10	32.9	10	32.3	10	57.4	66	49.4	50	15.8	20.8
**31931A**	K28	75.9	59	36.2	48	34.5	59	30.1	58	32.1	10	55.2	55	46.9	93	15.4	20.5
**32482**	K30	155	10	21.6	10	20.4	10	17.8	10	17.8	10	41.9	567	17.6	1620	15.5	19.8
**11078**	K34	17.9	10	17.8	10	19.3	10	17.7	10	18.3	10	89.9	10	19.7	10	15.6	15
**17390**	K34	4.61	10	16.8	10	4.9	10	4.88	10	4.96	10	44.7	10	5.12	10	4.18	14.4
**15201**	K34	4.86	10	17	10	5.13	10	5.34	10	5.2	10	63.1	10	5.5	10	4.45	14.7
**29002**	K34	32.1	10	14.9	10	34.1	10	34.2	10	31.3	10	357	10	36.2	10	21.5	12.5
**651**	KL39	4.78	50	15	36	5.03	87	6.14	10	3.64	10	6.81	96	16.9	10	4.02	13.3
**8422**	K39	4.66	10	14.6	10	4.84	57	6.13	52	4.38	10	6.29	10	16.6	10	3.85	13.2
**8629**	K39	5.03	56	15.2	35	5.26	55	7.7	58	5.03	28	8.48	43	25.8	10	4.15	13
**11034**	K39	4.69	58	16.3	52	5.05	44	6.65	10	4.9	33	6.94	89	17.4	118	4.11	14.1
**26702**	K39	5.01	10	15.6	103	4.91	40	6.81	61	4.64	59	6.87	63	17.7	61	4.11	13.7
**17355B**	K39	5.11	59	15.4	49	5.18	44	6.96	10	4.8	48	7.01	19	19.5	109	4.05	13.5
**2143A**	K39	5.52	51	16.6	10	5.59	10	7.86	10	5.62	10	8.07	60	18.8	10	4.12	13
**10340**	K39	29.3	10	15.2	51	28.9	10	38.7	59	26.9	52	37.1	10	103	10	22.9	12.8
**4603**	K39	27.9	57	23.1	10	19.5	10	28.5	10	18.8	10	25.6	10	44	10	14.9	20.1
**25004**	K39	86.2	58	16.1	51	32.7	52	90.9	49	29.7	51	41	10	44.4	10	23.5	14.2
**29986**	K39	30.4	10		10	26.2	10	34.1	10	25.3	10	33.9	10	65.6	10	21.6	12.4
**17681-1**	K62	29.7	10	14.7	10	91	10	2458	10	30	10	29	10	29	10	25.5	14
**17681-2**	K62	30.8	10	14.6	10	99	10	2530	10	32	10	30	10	30	58	25.1	13.2
**20863**	K62	79.5	27	14.5	21	97	10	2152	212	35	10	40	59	36	45	24.7	12.8
**21997**	K62	33.5	10	13.7	10	96	55	2228	10	35	10	37	10	34	10	27.1	13.6
**8094**	K62	27.1	10	14.5	10	108	10	2380	10	32	10	36	10	33	10	23.5	12.7
**8645**	K62	23.1	10	13.5	10	115	10	3640	249	26	10	22	10	23	10	19.5	12.7
**31876**	K62	21.2	81	12.9	20	77	10	2062	133	25	10	22	51	22	10	20.1	12
**8496A**	K62	22.3	10	15.9	64	64	41	1252	10	25	10	24	10	23	10	19.5	12.1
**9818**	K62	22.9	10	13.1	52	77	58	1825	260	27	55	25	51	23	47	20	12.2
**29774**	K64	50.9	56	42.0	10	60.7	10	49.2	10	57.2	10	68.4	73	44.5	60	23.7	25.2
**11522**	K64	20.7	59	22.4	59	21.1	39	32.8	61	5225	2793	21.7	58	22.2	10	19.3	20.3
**33058**	K64	21.5	64	23.3	63	20.6	69	28.1	10	3539	132	21	10	21.4	10	19.1	21.4
**18936**	K64	30.5	10	23.1	10	21.1	59	46.9	53	8888	599	21.2	10	21.7	110	19.8	21.7
**8139**	KL102	68.7	10	327.0	66	43.3	10	64.2	10	32.9	10	8789	3271	376	10	20.6	20.7
**10004**	KL102	84	10	337.0	45	43	10	78.7	10	35.5	10	8616	19802	362	10	21.9	22.2
**12641B**	KL102	25.9	10	491.0	38	29.8	10	26.2	10	25.6	10	4158	3916	132	10	19.6	25.3
**17814**	KL102	26	10	185.0	10	30.4	10	27.9	10	28.4	10	5071	2395	196	10	20.5	19.7
**2051**	KL102	55.6	10	348.0	33	37	10	47.7	10	31	10	6200	1070	236	10	21.1	20.5
**4415**	KL102	60.6	10	298.0	23	37.6	10	56.1	10	30.9	10	7882	1054	284	10	21	21.1
**17648**	KL105	124	10	84.4	10	97.7	10	74.3	10	56.7	10	2757	10	110	479	23.4	13.3
**16508**	KL107	58.3	43	16.1	10	20.8	55	37	10	20.8	40	23.1	10	31.6	10	19.2	12.4
**34831**	KL107	88.7	24	16.7	10	46.9	10	72.3	10	44.7	10	85.7	10	57	10	20.1	11.8
**923**	KL112	308	10	22.2	10	40.4	10	52.6	10	28.2	10	79.9	10	2751	1655	20.2	13.2
**4665**	KL112	33.9	10	21.0	192	37.3	269	31.3	10	34.4	60	38.6	10	34.2	10	22.2	12.3
**7789**	KL112	299	56	21.6	10	30.5	21	54.5	10	24.7	191	75.9	10	2829	10	19.1	13.6
**7939**	KL112	289	60	24.1	53	38.7	51	53	23	28.2	130	87.2	21	2795	70	19.1	12.6
**8871**	KL112	273	10	21.2	10	33.6	10	50.4	10	27.2	10	92.5	10	2761	190	19.2	12.6
**11938**	KL112	293	59	22.3	10	36.5	10	49.1	10	26.4	10	83.5	10	2652	10	19.2	12.8
**17247**	KL112	279	10	24.2	10	47.4	10	55	10	29.7	10	140	10	2938	1635	20	13
**19679**	KL112	329	10	22.0	10	33.3	10	55.8	10	27.7	10	85.8	10	3652	10	20.8	13.7
**20013**	KL112	189	10	24.0	10	25.6	10	50	10	29.4	10	101	10	3490	543	20.8	14.1
**22344**	KL112	216	10	25.5	10	35.8	10	49.9	10	28.1	10	79.6	10	2562	10	19.5	14.2
**23695**	KL112	174	10	24.2	10	29.9	10	44.2	10	24.7	10	87.8	10	3148	10	19.7	14
**25554**	KL112	252	10	27.5	10	32.9	10	47.2	10	25.8	10	75.8	10	2768	10	21.3	14.8
**32428**	KL112	37.4	10	32.1	10	42.2	10	34.9	10	36.2	10	43.1	10	35.8	10	26.3	17.2
**34053**	KL112	249	10	25.3	10	34.4	10	53.5	10	27.1	10	88.7	10	3504	10	21.5	15.2
**21657A**	KL112	231	10	24.9	10	34.2	10	55.6	10	27.7	10	83.7	10	3222	10	21	14.6
**28749A**	KL112	33.3	36	26.2	32	39.2	63	31.5	10	34.2	10	42.8	55	35.2	10	23.9	15.1
**15434**	KL112	29.7	10	25.5	10	33	10	27.5	10	29.5	10	38.5	10	28.5	51	21.8	14.6
**20198**	KL112	293	10	25.8	10	35.8	10	53.9	10	28	10	92	10	2732	10	21.4	15
**30405**	KL128	32.3	49	36.1	43	170	60	25.9	61	24.4	54	34.7	10	28.4	60	20.3	14.2

Supplementary Table 4 | Binding by FC and SBA of selected OAg sera against a panel of Kp isolates.

			anti O1-v1		anti O1-v2		anti O2-v1		anti O2-v2		
Strains	Genomic prediction	Chemical determination	MFI FC	SBA	MFI FC	SBA	MFI FC	SBA	MFI FC	SBA	neg CTRL MFI
5765B	O1	Gal	529	54	618	10	113	67	123	10	24.4
7008B	Untypeable	O1v2	2120	10	3104	10	160	10	398	10	24.4
28111	O4	O1v2	3409	10	3701	10	186	10	489	10	22.9
3037	O1	Gal	675	55	648	10	188	10	155	10	19.6
23485	O2a	Gal	156	73	39.1	75	6423	10	48.3	10	22.6
23415	O2a	Gal	2993	65	2049	10	121	10	254	10	20.3
27599	O1	Gal	418	10	332	10	127	23	64.9	10	18.9
11610	No genome	Gal	25	10	25.5	10	41	10	27.6	10	12.9
1092	O1	Gal	298	72	158	370	70	33	68.8	199	20.5
12993	O2a	Gal	84.3	43	33.5	32	407	1678	50.2	35	21.1
27152	O1	Gal	188	10	111	10	63.5	10	58.7	10	21.9
1637	O2a	Gal	72.2	57	35.7	33	164	1722	44.5	10	21.8
9098	O2a	Gal	58.6	114	24.8	10	461	5223	31.8	10	20.8
10319	O2a	Gal	69.3	54	38.3	42	219	3100	39.6	10	21.1
16698	O2a	Gal	59.8	51	35	10	293	1817	34	34	20.8
31931A	O2a	Gal	63.1	45	34.9	56	159	2381	40.7	45	20.5
32482	O1	Gal	755	10	467	10	35.8	10	66.4	10	19.8
11078	O2afg	Gal	18.6	10	41.3	62	99.8	426	140	432	15
17390	O2afg	Gal	17.5	10	37	55	118	222	145	316	14.4
15201	O2afg	Gal	18.2	10	33.4	55	82.8	331	103	406	14.7
29002	O2afg	Gal	15.9	10	38.4	60	94.8	273	139	354	12.5
651	O1	Gal	183	10	139	10	20	30	35.3	78	13.3
8422	O1	Gal	129	32	92.6	10	18	10	24.3	10	13.2
8629	O1	Gal	478	4678	326	10	23.8	84	54.7	154	13
11034	O1	Gal	48.6	10	46.7	10	21.6	70	21.6	118	14.1
26702	O1	Gal	92.9	10	88.2	10	22	22	24	110	13.7
17355B	O1	Gal	66.8	1465	63.2	10	20.8	65	22.1	10	13.5
2143A	O1	Gal	56.5	10	56.9	10	21.6	21	19.2	10	13
10340	O1	Gal	70.3	164	63.3	3583	18.4	10	21.4	10	12.8
4603	No genome	Gal	298	10	209	10	33.5	10	49.7	10	20.1
25004	O2afg	Gal	108	10	94.3	10	20.7	55	24.1	203	14.2
29986	O1	Gal	117	10	399	10	41.4	10	70	10	12.4
17681-1	O1	Gal	66	10	77.6	10	18.5	10	19	10	14
17681-2	O1	Gal	218	10	89	10	21.7	10	23.6	10	13.2
20863	O1	Gal	783	10	478	10	64.1	40	46.6	10	12.8
21997	O1	Gal	861	10	411	54	107	10	42.7	10	13.6
8094	O1	Gal	1266	10	980	10	31.1	10	93.4	10	12.7
8645	O1	Gal	1287	10	750	10	24.4	10	79	72	12.7
31876	O1	Gal	1006	10	574	1648	30.6	111	35.3	164	12
8496A	O1	Gal	828	10	516	1587	34.5	10	71.4	70	12.1
9818	O1	Gal	776	2856	121	1658	30.9	129	46.6	169	12.2
29774	O1	Gal	5330	10	2690	10	146	10	293	10	25.2
11522	No genome	Gal	107	10742	74.2	10	23.6	10	28.2	58	20.3
33058	O1	Gal	89	10	38.8	10	19.1	10	26.5	55	21.4
18936	O1	Gal	567	2018	360	1689	54.9	158	51.9	115	21.7
8139	O2afg	Gal	28.5	10	41.7	166	157	1009	101	1737	20.7
10004	O2afg	Gal	30.5	10	46.1	50	168	519	116	586	22.2
12641B	O2afg	Gal	36.2	10	46.3	23	163	382	84.9	864	25.3
17814	O2afg	Gal	24.2	10	35	10	200	398	81.9	160	19.7
2051	O2afg	Gal	30.2	10	47	32	155	206	114	495	20.5
4415	O2afg	Gal	28.6	10	44	10	150	185	120	213	21.1
17648	O2afg	Gal	33.1	10	55	62	305	1222	199	637	13.3
16508	O1	Gal	759	10	519	10	36.5	10	101	10	12.4
34831	O2afg	Gal	16.3	10	185	10	687	10	816	10	11.8
923	O1	Gal	1342	10	872	10	57.4	10	92.1	10	13.2
4665	O1	Gal	2319	512	1569	10	46.2	10	169	58	12.3
7789	O1	Gal	893	10	1175	10	101	10	93.5	10	13.6
7939	O1	Gal	597	1583	1137	10	136	10	135	54	12.6
8871	O1	Gal	1176	10	848	10	88.7	10	81.6	10	12.6
11938	O1	Gal	1141	10	930	10	57.7	10	88.5	10	12.8
17247	O1	Gal	490	10	1015	10	169	10	113	10	13
19679	O1	Gal	300	10	123	10	27.4	10	33.7	10	13.7
20013	O1	Gal	1349	10	998	481	92	10	118	10	14.1
22344	O1	Gal	1721	10	1282	10	31.1	10	137	10	14.2
23695	O1	Gal	1821	10	1342	10	57	10	132	10	14
25554	O1	Gal	9.65	10	1550	10	131	10	182	10	14.8
32428	O1	Gal	4041	10	2692	10	122	10	278	10	17.2
34053	O1	Gal	1864	10	8.69	10	116	10	146	10	15.2
21657A	O1	Gal	1670	10	1281	10	73.4	10	128	10	14.6
28749A	O2afg	Gal	1363	177	1713	10	122	10	246	10	15.1
15434	O1	Gal	3537	10	15.5	10	150	10	256	10	14.6
20198	O1	Gal	1866	10	1355	10	82.2	10	161	10	15

			anti O3 (long)		anti O3b (short)		anti O5		
Strains	Genomic prediction	Chemical determination	MFI FC	SBA	MFI FC	SBA	MFI FC	SBA	neg CTRL
**4998**	QC fail	Gal	13.4	10	39.5	10	15.7	10	11.9
**5948**	Untypeable	Gal	14.5	54.99	16.9	10	15.9	10	12.9
**29867**	O5	Man	16.5	54	21	20	461	1694	13.3
**1217**	O3/O3a	Man	121	10	45.4	10	29.4	10	11.8
**7703**	O3/O3a	Man	120	10	63.2	10	26	58	12.6
**25019**	O3/O3a	Man	64.1	10	27	10	20.2	10	11.8
**22011**	O3/O3a	Man	1138		217		14.6		12.1
**31154**	O3b	Man	210		485		15.3		11.5
**16666**	O3b	Man	54.4		707		16		11.8
**24649**	O3b	Man	295		406		13.4		12.2
**30405**	O3b	Man	340	5430	600	1786	21.8	47	12.3

			anti O4		
Strains	Genomic prediction	Chemical determination	MFI FC	SBA	neg CTRL
**1575**	O4	O4	424	683	12.3
**3890**	O4	O4	442	550	11.8
**7648**	O4	O4	404	540	12
**13647**	O4	O4	296	571	11.9
**14427**	O4	O4	582	578	12.1
**21120**	O4	O4	277	613	12.1
**25827**	O4	O4	608	526	11.7
**15383A**	No genome	O4	567	576	11.7
**15646B**	O4	O4	305	221	12.1
**16960A**	O4	O4	392	564	12
**24767-2**	O4	O4	408	610	11.6
**10988**	No genome	No OAg detected	18.3	10	12.1
**1015**	O4	O4	427	561	12.2
**6560**	O4	O4	442	683	12.2
**12200**	O4	O4	14.3	917	12.2
**13447**	O4	O4	480	1219	13.2
**24847**	O4	O4	330	608	12.5
**28111**	O4	O1v2	27.4	10	13.5
**28516**	O4	O4	11.3	556	13.5
**31381**	O4	O4	461	582	11.9
**31132A**	O4	O4	478	602	12.3

Supplementary Table 5 | IC50 from L-SBA performed with selected strains by testing different % of BRC. Positive standard sera and pre-immune sera were tested. IC50 was equal to 10 (no killing) with pre-immune sera in all tested conditions.

Isolate	IC50 (% BRC selected)	IC50 (Lower % BRC tested)		
KP88 (K62)	260 (30)	97 (20)	10 (10)	10 (5)
KP122 (K112)	1655 (50)	840 (30)	209 (20)	123 (10)
KP260 (K2)	912 (50)	431 (30)	187 (20)	10 (10)
